# Experimental demonstration of topological bounds in quantum metrology

**DOI:** 10.1093/nsr/nwae065

**Published:** 2024-02-26

**Authors:** Min Yu, Xiangbei Li, Yaoming Chu, Bruno Mera, F Nur Ünal, Pengcheng Yang, Yu Liu, Nathan Goldman, Jianming Cai

**Affiliations:** School of Physics, Hubei Key Laboratory of Gravitation and Quantum Physics, Institute for Quantum Science and Engineering, Huazhong University of Science and Technology, Wuhan 430074, China; International Joint Laboratory on Quantum Sensing and Quantum Metrology, Huazhong University of Science and Technology, Wuhan 430074, China; School of Physics, Hubei Key Laboratory of Gravitation and Quantum Physics, Institute for Quantum Science and Engineering, Huazhong University of Science and Technology, Wuhan 430074, China; International Joint Laboratory on Quantum Sensing and Quantum Metrology, Huazhong University of Science and Technology, Wuhan 430074, China; School of Physics, Hubei Key Laboratory of Gravitation and Quantum Physics, Institute for Quantum Science and Engineering, Huazhong University of Science and Technology, Wuhan 430074, China; International Joint Laboratory on Quantum Sensing and Quantum Metrology, Huazhong University of Science and Technology, Wuhan 430074, China; Advanced Institute for Materials Research (WPI-AIMR), Tohoku University, Sendai 980-8577, Japan; TCM Group, Cavendish Laboratory, University of Cambridge, Cambridge CB3 0HE, UK; School of Physics, Hubei Key Laboratory of Gravitation and Quantum Physics, Institute for Quantum Science and Engineering, Huazhong University of Science and Technology, Wuhan 430074, China; International Joint Laboratory on Quantum Sensing and Quantum Metrology, Huazhong University of Science and Technology, Wuhan 430074, China; International Joint Laboratory on Quantum Sensing and Quantum Metrology, Huazhong University of Science and Technology, Wuhan 430074, China; Institut für Theoretische Physik and IQST, Universität Ulm, Ulm D-89081 Germany; Center for Nonlinear Phenomena and Complex Systems, Université Libre de Bruxelles, Brussels B-1050, Belgium; Laboratoire Kastler Brossel, Collège de France, Paris 75005, France; School of Physics, Hubei Key Laboratory of Gravitation and Quantum Physics, Institute for Quantum Science and Engineering, Huazhong University of Science and Technology, Wuhan 430074, China; International Joint Laboratory on Quantum Sensing and Quantum Metrology, Huazhong University of Science and Technology, Wuhan 430074, China; Shanghai Key Laboratory of Magnetic Resonance, East China Normal University, Shanghai 200062, China

**Keywords:** quantum metrology, multi-parameter estimation, topological bounds, Berry curvature, Chern number, topological phase transition

## Abstract

Quantum metrology is deeply connected to quantum geometry, through the fundamental notion of quantum Fisher information. Inspired by advances in topological matter, it was recently suggested that the Berry curvature and Chern numbers of band structures can dictate strict lower bounds on metrological properties, hence establishing a strong connection between topology and quantum metrology. In this work, we provide a first experimental verification of such topological bounds, by performing optimal quantum multi-parameter estimation and achieving the best possible measurement precision. By emulating the band structure of a Chern insulator, we experimentally determine the metrological potential across a topological phase transition, and demonstrate strong enhancement in the topologically non-trivial regime. Our work opens the door to metrological applications empowered by topology, with potential implications for quantum many-body systems.

## Teaser

This work bridges topology and quantum metrology, demonstrating how topological characteristics within band structures, namely Berry curvature and Chern number, set fundamental limits on metrological properties, with experimental validation in an emulated Chern insulator.

## INTRODUCTION

Exploring the limit of the quantum precision measurement, as governed by the laws of quantum mechanics, not only leads to disruptive applications in quantum enhanced metrology [1–8], but also provides novel insights into fundamental concepts in quantum physics, such as entanglement, nonlocality and criticality [9–15]. The precision limit for single-parameter estimation is given by the quantum Cramér–Rao bound (CRB) [1], which relates the best achievable measurement precision to the inverse of the quantum Fisher information (QFI) of the underlying quantum state. From a geometric perspective, the quantum Cramér–Rao bound set by the QFI for single-parameter estimation is connected to the quantum metric [1,9], which has recently been the focus of increased attention due to the recently established connection to flatband superconductivity [16,17]. This geometric property of quantum states corresponds to the real part of the quantum geometric tensor [18,19], which was also recently measured in experiments [20–24].

More importantly, the imaginary part of the quantum geometric tensor corresponding to the Berry curvature plays a central role in topological physics, e.g. in quantum Hall-type transport [25,26] and topological defects [27]. Surprisingly, inspired by the existence of correlations between the quantum metric and the Berry curvature, it has been suggested that the Berry curvature (and the related Chern numbers) can set topological bounds on quantum multi-parameter estimation [28,29]. Therefore, demonstrating the fundamental connection between topology and quantum metrology in experiments is highly appealing. While recent experiments realized and verified the CRB through QFI measurements [30–35] in the context of single-parameter-estimation schemes [35], the extension to multi-parameter scenarios is generally more complex and challenging due to the possible incompatibility of optimal quantum measurements for each individual parameter [36–46]. Accessing the limits of quantum multi-parameter estimation has remained elusive, and thus the experimental demonstration of topological bounds in quantum metrology has never been explored.

In this work, we address these challenges and present the first experiment connecting multi-parameter metrological bounds to topological band structures, using a synthetic topological system emulating a two-dimensional Chern insulator. By performing optimized positive operator-valued measurements (POVMs) to implement quantum multi-parameter estimation of this synthetic topological system, we obtain the best achievable measurement precision. This allows us to experimentally verify the metrological bound given by the Berry curvature, and, more importantly, saturate the Holevo bound pertaining to geometric properties of the system. The developed techniques enable us to characterize quantum metrological potential across different topological regimes, which exhibits an appealing connection to the Chern number. Our results pave the way for considerations beyond the single-particle case, where the fundamental connection between quantum metrology, the Berry curvature and the Chern numbers of band structures is anticipated to have an important impact in many-body settings with the precision of multi-parameter estimation dictated by the underlying topology.

## QUANTUM MULTI-PARAMETER ESTIMATION OF A SYNTHETIC TOPOLOGICAL SYSTEM

General (and exact) relations between the quantum metric, the QFI and topological invariants exist for generic Dirac Hamiltonians in arbitrary spatial dimensions [28]. To experimentally investigate and verify these relations in detail, we utilize a nitrogen-vacancy (NV) center in diamond to implement a two-level synthetic topological system, which can describe a Chern insulator in two dimensions. The ground state of the NV center spin has three spin sublevels, *m_s_* = 0, ±1. By applying an external magnetic field along the NV axis, we lift the degeneracy of the spin states *m_s_* = ±1 and employ the spin sublevels *m_s_* = 0, −1 to encode the two-level Hamiltonian; the additional spin state *m_s_* = +1 is used for the implementation of POVM measurements (see Sec. C of the online supplementary material for more details). In the experiment, we use a home-built confocal setup to manipulate the NV center spin in diamond. A 532-nm green laser pulse is used to polarize and readout the spin state of the NV center. The external magnetic field is applied by a permanent magnet. The amplitude and phase modulation microwave pulses are firstly generated by an arbitrary waveform generator and then amplified by a microwave amplifier before being delivered to the sample through a copper line. An avalanche photodiode is used to collect the fluorescence emitted from the NV center spin. Our experiment aims at emulating the massive Dirac model [47,48], given by the Bloch Hamiltonian


**Figure 1.**
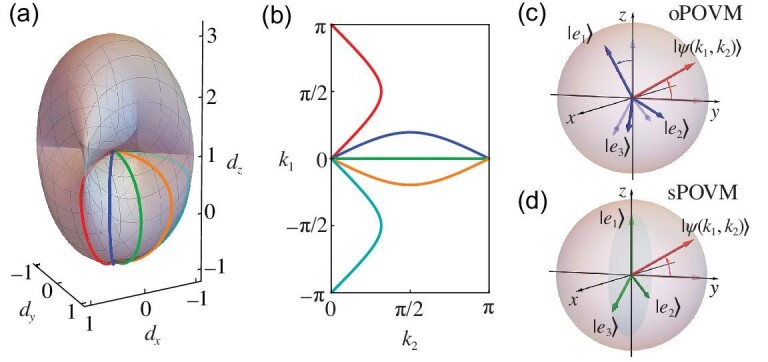
Topology of a two-band Chern insulator and POVM for quantum multi-parameter estimation. (a) Surface of the terminal points $\boldsymbol{d}(\boldsymbol{k})$ [cf. Equation (1) with *M* = 1]. (b) The curves of different colors correspond to different trajectories in the Brillouin zone $\boldsymbol{k}=(k_1,k_2)\in \mathbb {T}^2$. Panels (c)–(d) depict (c) the optimized POVM (oPOVM) for different states (blue arrows) and (d) a fixed set of symmetric POVM (sPOVM). The red arrows denote the Bloch vector of the excited state encoding the information of unknown parameters.

(1) \begin{eqnarray*}
H(\boldsymbol{k}) &=&\boldsymbol{d}_{\boldsymbol{k}}\cdot \boldsymbol{\sigma } \\
& =&\sum _{i=1}^2\sin (k_i)\sigma _i +\bigg (M-\sum _{i=1}^2\cos (k_i)\bigg )\sigma _3,\\
\end{eqnarray*}

where $\boldsymbol{d}_{\boldsymbol{k}}\in \mathbb {R}^3$ and $\boldsymbol{k}\in \mathbb {T}^2$ are quasimomenta. Here, $\mathbb {R}^3$ denotes the real coordinate three-dimensional space, and $\mathbb {T}^2$ represents the two-dimensional torus. This model describes the band structure of a two-band Chern insulator [Fig. 1(a)–(b)], exhibiting the quantum anomalous Hall effect. Away from the critical values of *M* where the system is gapless, the vector $\boldsymbol{d}_{\boldsymbol{k}}$ gives rise to a well-defined unit vector $\boldsymbol{n}_{\boldsymbol{k}}=\boldsymbol{d}_{\boldsymbol{k}}/|\boldsymbol{d}_{\boldsymbol{k}}|\in \mathcal {S}^2$, with $\mathcal {S}^2$ referring to the 2-sphere. The Hamiltonian in Equation (1) is associated with two bands, with opposite Chern numbers and Berry curvature $\Omega _{12}(\boldsymbol{k})$. The aim of this work is to emulate such a two-band Chern insulator and experimentally explore the connections between topology and multi-parameter estimation by performing the latter on a specific band. The parameters $\boldsymbol{k}=(k_1, k_2)$ represent the quasimomentum of the emulated system, and are the unknown parameters to be estimated. We remark that the determination of the quasimomentum is of relevance and significance for quantum many-body systems [49,50]. The principles and methods developed in our experiment, as well as the measurement of Berry curvature and the quantum metric may also have potential application in many-body systems, such as ultra-cold atoms [49,51,52] (see Sec. E of the online supplementary material) and exciton polariton (interacting photon) systems [53].

In our experiment, we estimate $\boldsymbol{k}$ by performing measurements on the eigenstates of a given band of the emulated system, in this case, the excited state $\vert \psi (\boldsymbol{k})\rangle$ of the Hamiltonian [see Equation (1)]. We first apply a 532-nm green laser pulse to initialize the NV center spin in the *m_s_* = 0 state. The subsequent microwave pulse $H_i(t)=A_1 \cos\! \left(\omega _1 t + 3\pi /2-\varphi _{\boldsymbol{k}} \right) (\mathinner {|{0}\rangle }\mathinner {\langle {-1}|}+\mathinner {|{-1}\rangle }\mathinner {\langle {0}|})$ over a duration of $(\theta _{\boldsymbol{k}}/A_1)$, rotates the spin around the axis $\hat{l}=(-\sin {\varphi _{\boldsymbol{k}}}, \cos {\varphi _{\boldsymbol{k}}}, 0)$ by an angle $\theta _{\boldsymbol{k}}$ and prepares the system in state $\vert \psi (\boldsymbol{k})\rangle$. Note that the angles $\theta _{\boldsymbol{k}}$ and phases $\varphi _{\boldsymbol{k}}$, as determined by the phase and duration of the microwave pulse, tune the quasimomentum parameters $\boldsymbol{k}=(k_1, k_2)$ of the emulated system according to the relation $\boldsymbol{n}_{\boldsymbol{k}}=\boldsymbol{d}_{\boldsymbol{k}}/|\boldsymbol{d}_{\boldsymbol{k}}|=(\sin {\theta _{\boldsymbol{k}}}\cos {\varphi _{\boldsymbol{k}}}, \sin {\theta _{\boldsymbol{k}}}\sin {\varphi _{\boldsymbol{k}}}, \cos {\theta _{\boldsymbol{k}}})$.

To extract the complete information of both components *k*_1_ and *k*_2_ from state $\vert \psi (\boldsymbol{k})\rangle$ (see Sec. B of the online supplementary material), a two-outcome projective measurement is not sufficient. One needs to implement a generalized quantum measurement (namely, a POVM), which can be specified by a set of operators as $\Pi =\lbrace \Pi _i\mid \sum _i\Pi _i=\hat{{1\!\!1}},\, \Pi _i\ge 0, \, i=1,\dots ,m\rbrace$ on the system with *m* ≥ 3 [38]. The results of *N* measurement repetitions are represented as $\vec{x}=(x_{1},x_{2},\dots ,x_{k},\dots ,x_{N})$, where $x_k\in \lbrace a_i\rbrace _{i=1}^m$ and *a_i_* denotes the measurement outcome corresponding to Π_*i*_. To optimally infer vector $\boldsymbol{k}$, we construct the maximum likelihood estimator $\hat{\boldsymbol{k}}$ from the probability estimators $\boldsymbol{\hat{p}}_\Pi (\vec{x})$, with $\boldsymbol{\hat{p}}_\Pi ^j(\vec{x}) =(1/N) \sum _{k=1}^N \delta _{a_j,x_{k}}$, by solving the likelihood equation (see Sec. D of the online supplementary material). Consequently, the covariance matrix $\Sigma (\hat{\boldsymbol{k}})$ of the maximum likelihood estimator $\hat{\boldsymbol{k}}$ can be obtained as


(2) \begin{eqnarray*}
\Sigma (\hat{\boldsymbol{k}}) =\bigg ( \frac{\partial \hat{\boldsymbol{k}}}{\partial \boldsymbol{\hat{p}}_\Pi } \bigg ) \Sigma (\boldsymbol{\hat{p}}_\Pi )\bigg ( \frac{\partial \hat{\boldsymbol{k}}}{\partial \boldsymbol{\hat{p}}_\Pi } \bigg )^\top ,
\end{eqnarray*}

where $\Sigma (\boldsymbol{\hat{p}}_\Pi )$ is the covariance matrix of $\boldsymbol{\hat{p}}_\Pi$ and $({\partial \hat{\boldsymbol{k}}}/{\partial \boldsymbol{\hat{p}}_\Pi })$ is the associated Jacobian matrix. The determinant of the covariance matrix $\mathrm{det}\Sigma (\hat{\boldsymbol{k}})$ is the generalized variance, which can be thought of as a measure of dispersion for the multivariate estimated parameters around the true value. A larger generalized variance means that the estimated data points are more spread out in the multi-dimensional space. The square root of the generalized variance, $[\mathrm{det} \Sigma (\hat{\boldsymbol{k}})]^{1/2}$, measures the overall dispersion of multiple parameters, which we refer to as the measurement uncertainty volume (MUV). This quantity is proportional to the volume of the hyper-elliptical estimated data cloud in $\hat{\boldsymbol{k}}$ space [54].

In our experiment, we adopt a set of three-element rank-1 POVM $\lbrace \Pi _i=\mathinner {|{e_i}\rangle }\mathinner {\langle {e_i}|},\, i=1,2,3\rbrace$ that allows us to construct an unbiased estimator for two unknown parameters simultaneously (see Sec. B of the online supplementary material for details). Such POVMs can be described with parameters *r_i_*, θ_*i*_ and ϕ_*i*_ by setting


(3) \begin{eqnarray*}
\mathinner {|{e_i}\rangle } = r_i \bigg ( \cos {\frac{\theta _i}{2}} \mathinner {|{0}\rangle } + \sin {\frac{\theta _i}{2}}e^{i\varphi _i} \mathinner {|{-1}\rangle } \bigg ).
\end{eqnarray*}

Note that the normalization condition of a POVM requires that $\sum _{i=1}^3 r_i^2=2$; thus, $\lbrace \mathinner {|{e_i}\rangle }\rbrace _{i=1}^3$ is a set of unnormalized non-orthogonal vectors in the two-dimensional Hilbert space. This POVM is realized through a projective measurement $\lbrace \mathinner {|{u_i}\rangle }\mathinner {\langle {u_i}|}\rbrace _{i=1}^3$ in the extended three-level Hilbert space, where $(\mathinner {|{0}\rangle }\mathinner {\langle {0}|}+\mathinner {|{-1}\rangle }\mathinner {\langle {-1}|})\mathinner {|{u_i}\rangle } = \mathinner {|{e_i}\rangle }$, by taking advantage of the auxiliary spin sublevel *m_s_* = +1 of the NV center. To achieve this goal, we first apply unitary transformations on the NV center, which rotate states $\mathinner {|{u_i}\rangle }$ to the spin sublevels $\lbrace \mathinner {|{0}\rangle },\mathinner {|{\pm 1}\rangle }\rbrace$ by engineering microwave driving fields on resonance with both transitions *m_s_* = 0 ↔ *m_s_* = ±1. The subsequent spin-dependent fluorescence measurement realizes projective measurements along the basis $\lbrace \mathinner {|{u_i}\rangle }\rbrace$, which is equivalent to the POVM $\lbrace \Pi _i=\mathinner {|{e_i}\rangle }\mathinner {\langle {e_i}|}\rbrace _{i=1}^3$ in the two-level Hilbert space spanned by $\lbrace \mathinner {|{0}\rangle },\mathinner {|{-1}\rangle }\rbrace$ (see Sec. C of the online supplementary material).

**Figure 2.**
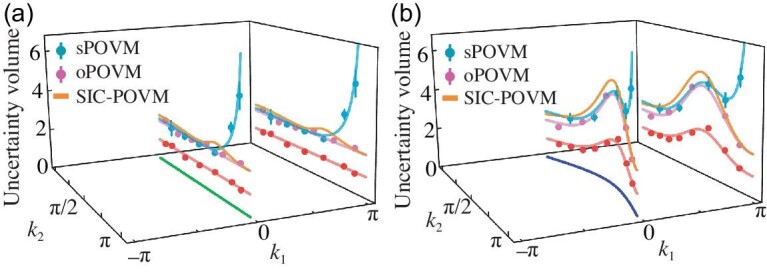
The MUV $[\mathrm{det} \Sigma (\hat{\boldsymbol{k}})]^{1/2}$ as quantified by the square root of the generalized variance along two different trajectories in $\boldsymbol{k}$ space, i.e. the green and blue curves [cf. Fig. 1(a)–(b)]. The oPOVM achieves better performance over the state-independent sPOVM and the SIC-POVM (theory), which are also compared with the bound given by the Berry curvature [the right-hand side of Equation (5); red dots]. The curves represent theoretical predictions. For better visibility, the curves and dots are projected to the side.

The above appropriate parameterization of the POVM [i.e. Equation (3)] enables us to identify and implement a simple POVM that maximizes the determinant of the corresponding Fisher information matrix, which we denote as oPOVM (see Sec. B of the online supplementary material for more details); see Fig. 1(c). In addition, we also implement a symmetric POVM (sPOVM) with $r_i=\sqrt{2/3}$, φ_*i*_ = 0 and θ_*i*_ = 0, ±2π/3; see Fig. 1(d). In Fig. 2, we display the MUV for the quantum multi-parameter estimation associated with the excited state $\mathinner {|{\psi (\boldsymbol{k})}\rangle }$ of the synthetic topological Hamiltonian in Equation (1). The experimental results are obtained by two different types of POVM measurements, namely, the optimized POVM and the symmetric POVM. Here, we construct the probability estimator $\boldsymbol{\hat{p}}_\Pi (\vec{x})$ from the experimental measurement outcomes, and obtain the covariance matrix $\Sigma (\hat{\boldsymbol{k}})$ according to Equation (2) (see Sec. D of the online supplementary material for details). For comparison, we also present the values that can be achieved via the symmetric, informationally complete POVM (SIC-POVM) [55–58], which represents the most versatile class of measurements to obtain information about the state of a quantum system. It can be seen from Fig. 2 that the optimized POVM that we identify indeed achieves better measurement performance over both the sPOVM and SIC-POVM.

## OPTIMAL QUANTUM MULTI-PARAMETER ESTIMATION AND TOPOLOGICAL BOUNDS

The above-developed techniques enable us to experimentally explore the metrological bounds related to the topology of the system. We note that the multi-parameter CRB (we refer to this bound as the quantum SLD-CRB in the following) establishes a lower bound for the covariance matrix [38] (see Sec. A of the online supplementary material):


(4) \begin{eqnarray*}
\Sigma (\hat{\boldsymbol{k}})\ge \frac{1}{N} \mathcal {F}^{-1}_{\mathbb {T}^2}.
\end{eqnarray*}

Here *N* represents the number of repeated measurements and $\mathcal {F}_{\mathbb {T}^2}$ is the QFI matrix of $\mathinner {|{\psi (\boldsymbol{k})}\rangle }$ with respect to vector $\boldsymbol{k}$. Remarkably, the Berry curvature $\Omega _{12}(\boldsymbol{k})$ associated with state $\mathinner {|{\psi (\boldsymbol{k})}\rangle }$ is related to the quantum metric [and thereby the QFI matrix as $\mathcal {F}_{\mathbb {T}^2} =4 g(\boldsymbol{k})$] through $[{\mathrm{det}(g(\boldsymbol{k})})]^{1/2}=|\Omega _{12}(\boldsymbol{k})|/2$. This surprisingly concise identification has important metrological implications: the uncertainty volume is bounded by the Berry curvature as [28]


(5) \begin{eqnarray*}
[\mathrm{det} \Sigma (\hat{\boldsymbol{k}})]^{1/2} > \frac{1}{2{\textit{N}}}\frac{1}{|\Omega _{12}(\boldsymbol{k})|}.
\end{eqnarray*}

In our experiment, we obtain the MUV $[\mathrm{det} \Sigma (\hat{\boldsymbol{k}})]^{1/2}$ achieved by the optimized POVM, and directly extract the Berry curvature using the method of weak parametric modulations [21,24] (see Sec. G of the online supplementary material). This allows us to directly compare the best achievable MUV with the Berry curvature bound [namely, the right-hand side of Equation (5)]. The results shown in Fig. 2(a)–(b) not only experimentally verify the Berry curvature’s role in defining the metrological capabilities of topological systems for the first time, but also suggest that the achieved optimal MUV shows strongly correlated behavior with the Berry curvature bound. This implies that a larger Berry curvature is associated with a better metrological performance (namely, a smaller MUV). A detailed physical explanation of this connection is provided in the online supplementary material Sec. A. Hence, our experiment demonstrates how extracting the Berry curvature—an effective magnetic field in momentum space [25]—provides a practical scheme to predict the metrological potential of topological band systems.

**Figure 3.**
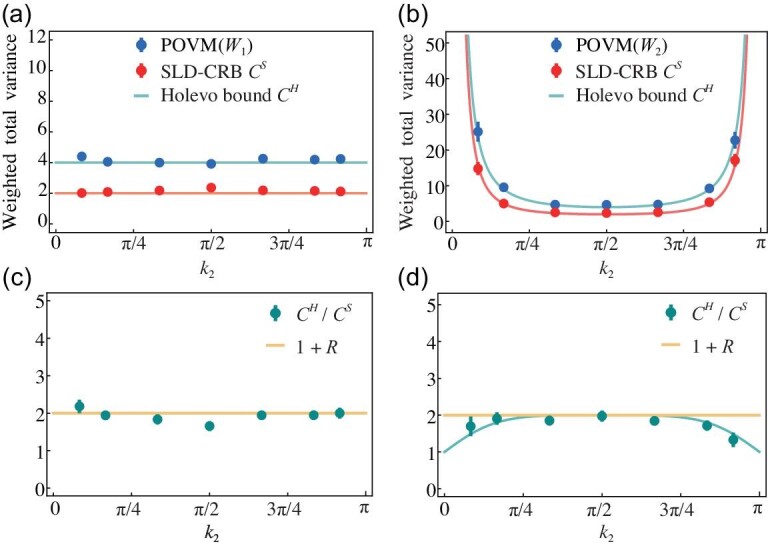
Scalar CRBs for quantum multi-parameter estimation, along a trajectory in $\boldsymbol{k}$ space, i.e. the green curve in Fig. 1(a)–(b), with respect to different weight matrices ${\textit W}_1= {\mathcal F}_{\mathbb {T}^2}$ (a) and ${\textit W}_2=J^\top J$ (b). POVM($\textit {W}_j$) (*j* = 1, 2), which is optimized to achieve the minimal value of $\mathrm{Tr}(\textit {W}_j {\boldsymbol F}_{\boldsymbol C}^{-1})$ for the Fisher information matrix *F_C_*, saturates the Holevo bound, $\textit {C}^H(\boldsymbol{k},\textit {W}_j)$. Panels (c)–(d) show the ratio between the Holevo bound and the SLD-CRB $(\textit {C}^H/\textit {C}^S)$ for the weight matrices $\textit {W}_1$ (c) and $\textit {W}_2$ (d), which is compared with the characterization parameter $1+\textit {R}$. The curves represent theoretical predictions.

In addition to the MUV, the precision for multi-parameter estimation is characterized by the weighted total variance $\mathrm{Tr}(\it {W}\Sigma (\hat{\boldsymbol{k}}))$ with a positive real weight matrix $\it {W}$. Although the quantum SLD-CRB is generally achievable in the case of single-parameter estimation, its multi-parameter version is not always attainable due to the fundamental incompatibility between optimal measurement operators associated with different parameters [38] (see also Sec. A of the online supplementary material). The achievable measurement precision limit as quantified by the weighted total variance is given by the Holevo bound (referred to as the attainable quantum CRB) [59] (see Sec. A of the online supplementary material), which can only be obtained as an optimization. The techniques that we develop for the optimization and implementation of the POVM allow us to achieve such a non-trivial goal. In the experiment, for a chosen weight matrix $\it {W}_j$, we perform the optimized POVM, the corresponding Fisher information matrix (*F_C_*) of which minimizes the value of $\mathrm{Tr}(\it {W}_j {\textit{F}}_C^{-1})$, and obtain the covariance matrix $\Sigma (\hat{\boldsymbol{k}})$. As demonstrated in Fig. 3(a), when we choose the weight matrix ${\it {W}}_1=\it\mathcal {F}_{\mathbb {T}^2}$, the achieved scalar measurement uncertainty $\mathrm{Tr}({\it {W}}_1 \it\Sigma (\hat{\boldsymbol{k}}))$ indeed reaches the corresponding Holevo bound ${C}^H(\boldsymbol{k},{\it {W}}_1)$. This weight matrix can accurately characterize the difference between the estimated and the true states. As a second example, we consider the weight matrix ${\it {W}}_2=\it J^{T}J$, where *J* is the Jacobian matrix associated with the pullback map from the 2-sphere $\mathcal {S}^2$ to the Brillouin zone $\mathbb {T}^2$ [28] (see Eq. (S.60) and Sec. B of the online supplementary material). Similarly, we perform the optimized POVM with respect to the weight matrix ${\it {W}}_2$, which also saturates the corresponding Holevo bound [Fig. 3(b)]. We remark that the attainability of the quantum SLD-CRB and the Helovo bound is considered in a local sense [38,59,60]. This means that one usually has sufficient prior knowledge of the parameters, ensuring that they are in close proximity to a known value $\boldsymbol{k}_0$. Therefore, both the optimized POVM and the weight matrix are actually determined with reference to $\boldsymbol{k}_0$.

Remarkably, the Holevo bound has significant geometric relevance [29,61,62], and is connected (via the Berry curvature) to the quantum SLD-CRB ${\it C}^S(\boldsymbol{k},\it {W})\equiv \mathrm{Tr}({\it {W} \mathcal {F}}_{\mathbb {T}^2}^{-1})$, namely, ${\it C}^H(\boldsymbol{k},{\it {W}})\le (1+{\it R}){\it C}^S(\boldsymbol{k},\it {W})$ [29,37,63]. The parameter ${\it R}= \Vert i2\mathcal {F}_{\mathbb {T}^2}^{-1}\Omega \Vert _{\infty }\in [0,1]$ is related with the Berry curvature Ω, with ‖ · ‖_∞_ taking the largest eigenvalue [29]. We determine the quantum SLD-CRB ${\it C}^S(\boldsymbol{k},\it {W})$ for the weighted total variance by measuring the quantum metric $\sim \mathcal {F}_{\mathbb {T}^2}/4$ [33] (see Sec. G of the online supplementary material). The results in Fig. 3(c)–(d) show the ratio between the Holevo bound and the quantum SLD-CRB ${\it C}^{H}(\boldsymbol{k},\it {W})/{\it C}^S(\boldsymbol{k},\it {W})$ and directly testify the attainability of the quantum SLD-CRB. We remark that the Berry curvature bound and the attainability of the quantum SLD-CRB (as we metrologically characterize in experiments) reveal the intriguing role of Berry curvature in determining metrological potential of topological systems: a larger Berry curvature would be beneficial for the measurement precision of quantum multi-parameter estimation; however, it may indicate a weaker attainability of the quantum SLD-CRB.

## METROLOGICAL CHARACTERIZATION OF TOPOLOGICAL BANDS

Furthermore, we experimentally explore the metrological potential of the Bloch Hamiltonian [Equation (1)] in different topological regimes governed by the control parameter *M*, where |*M*| > 2 and |*M*| < 2 correspond to topologically trivial (with the Chern number Ch_1_ = 0) and non-trivial (Ch_1_ = 1) cases, respectively. The quantum volume of the momentum space, which is sensitive to the topology, is defined as $\mathrm{vol}_g (\mathbb {T}^2)\equiv \int _{\mathbb {T}^{2 }} [\mathrm{det}(g(\boldsymbol{k}))]^{1/2}\mathrm{d} \boldsymbol{k}$ [28].

**Figure 4.**
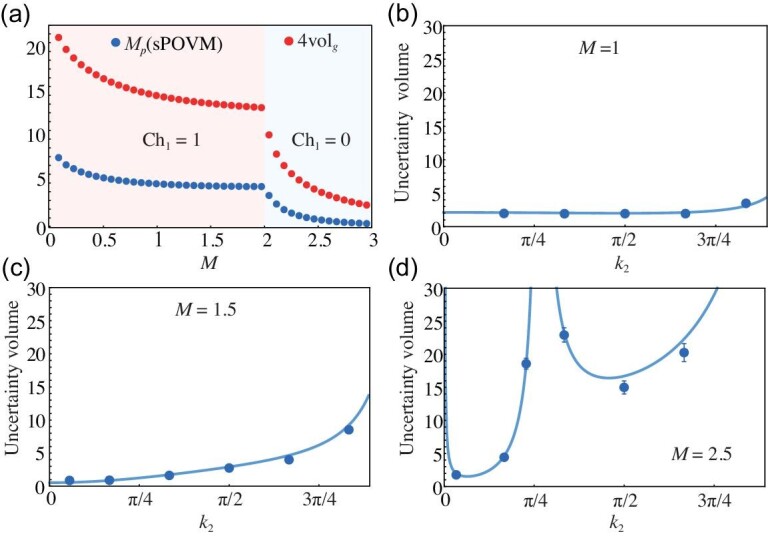
(a) Quantum volume $4\mathrm{vol}_g (\mathbb {T}^2)$ is compared with the integration of the inverse of the uncertainty volume ${\textit M}_p\equiv (1/N)\int _{\mathbb {T}^{2 }} [\mathrm{det} \Sigma (\hat{\boldsymbol{k}}) ]^{-1/2} \mathrm{d} \boldsymbol{k}$ by the sPOVM. Panels (b)–(d) show the MUV $[\mathrm{det} \Sigma (\hat{\boldsymbol{k}}) ]^{1/2}$ obtained by the sPOVM along a trajectory in $\boldsymbol{k}$ space [i.e. the green curve in Fig. 1(a)–(b)] for topologically different regimes. The blue dots represent experimental data, while theoretical values are indicated with lines.

In Fig. 4(a), we present the integrated MUV, ${\it M}_p \equiv (1/N)\int _{\mathbb {T}^{2 }} [\mathrm{det} \Sigma (\hat{\boldsymbol{k}}) ]^{-1/2} \mathrm{d}{\textit{k}}_1 \mathrm{d}{\textit{k}}_2$, over the Brillouin zone for the protocol of a specific POVM, e.g. the symmetric one. In the theory of global sensing [60], this quantity, serving as a metric for the average performance of parameter estimation when prior information of the parameters to be estimated is lacking, shows correlated behaviour with the quantum volume across the topological transition at *M* = 2. Our result experimentally confirms that the quantum volume can predict the metrological potential of a topological system, namely, a larger quantum volume $\mathrm{vol}_g (\mathbb {T}^2)$ implies a better global metrological performance in the Brillouin zone. We remark that the integration of the metric–Berry curvature relation over the Brillouin zone links the quantum volume $\mathrm{vol}_g (\mathbb {T}^2)$ to a topological invariant, namely, the first Chern number Ch_1_ of the associated Bloch band. This relation further predicts that the system’s metrological performance may strongly depend on its topological invariants, as illustrated by Fig. 4(a).

We proceed to choose *M* = 1 and *M* = 1.5 in the topologically non-trivial regime and *M* = 2.5 in the topologically trivial regime, and experimentally determine the MUV to illustrate the corresponding metrological performance; see Fig. 4(b)–(d). Notably, in the topologically trivial regime, as depicted in Fig. 4(d), the uncertainty volume exhibits a significant variation with *k*_2_ around *k*_2_ = π/4. This behavior stems from the Berry curvature becoming zero at this point (see Fig. S.5 and Sec. F of the online supplementary material for details). These results clearly demonstrate the contrast in metrological potentials of the topologically different regimes: the MUV is significantly smaller in the topologically non-trivial regime (Ch_1_ = 1) than in the topologically trivial regime (Ch_1_ = 0). Hence, our experiment provides clear evidence that topology influences metrological potential in a non-trivial way.

## EXTENSION TO A MANY-BODY SYSTEM

In the above sections, the parameters to be estimated are the quasimomenta in the Bloch Hamiltonian. Here we extend the metrology scenario to the many-body system by considering a two-band Chern insulator on a torus geometry. By threading two magnetic fluxes ϕ_1, 2_ through two independent non-contractible cycles of the torus, one obtains a family of many-body ground states over ϕ_1, 2_ space [64,65]. Because the ground state of the system depends on ϕ_1, 2_, one can estimate the magnetic fluxes ϕ_1, 2_. The MUV $\sqrt{\mathrm{det}(\Sigma (\phi _1, \phi _2))}$ for determining the magnetic flux ϕ_1, 2_ is bounded by $\sqrt{\mathrm{det}(\mathcal {F}(\phi _1, \phi _2))}$, where $\mathcal {F}(\phi _1, \phi _2)$ is the quantum Fisher information matrix that, in the thermodynamic limit, is expressed in terms of the momentum-space quantum metric as


(6) \begin{eqnarray*}
\mathcal {F} (\phi _1, \phi _2) = \frac{1}{\pi ^2} \int _{\mathbb {T}^{2 }} g(\boldsymbol{k}) \mathrm{d} \boldsymbol{k}.
\end{eqnarray*}

Then we can find that [66] $\sqrt{\mathrm{det}(\mathcal {F} (\phi _1, \phi _2))} \ge \mathrm{vol}_g (\mathbb {T}^2) / {\pi ^2}\ge |\mathrm{Ch}_{1}| / {\pi }$. The above relation connects the metrological performances for estimating the magnetic flux and the system’s topological properties. According to the relationship between the metrological potential ${\it M}_p$ and the quantum volume $\mathrm{vol}_g (\mathbb {T}^2)$, we also have $\sqrt{\mathrm{det}(\mathcal {F} (\phi _1, \phi _2))} \ge \mathrm{vol}_g (\mathbb {T}^2) / {\pi ^2} \ge {\it M}_p /{(4\pi ^2)}$. Therefore, the relation between metrological performances and topology revealed above can be extended to such a scenario, which connects the estimation of magnetic fluxes and topology.

## DISCUSSION AND CONCLUSION

Our experiment demonstrates how the quasimomentum $\boldsymbol{k}=(k_1,k_2)$, encoded in the NV spin state $\mathinner {|{\psi (\boldsymbol{k})}\rangle }=\cos {[{\theta (\boldsymbol{k})}/{2}]}\mathinner {|{0}\rangle } +\sin [{{\theta (\boldsymbol{k})}/{2}}]e^{i \varphi (\boldsymbol{k})}\mathinner {|{-1}\rangle }$ is estimated using an optimized POVM. This protocol offers a promising opportunity to enhance the accuracy of measurements for various physical quantities utilizing NV centers. By initializing the NV center to a specific state $\mathinner {|{\psi _0}\rangle }$ and letting it evolve for a time *t*_0_ under the NV center’s spin Hamiltonian [67]


(7) \begin{eqnarray*}
&&H(\boldsymbol{\lambda }) = D \bigg ( S_z^2 - \frac{2}{3} \bigg ) + \gamma \boldsymbol{B} \cdot \boldsymbol{S} \\ &&\quad \quad +\, \rm {electric \,\,interaction} + \rm {other \,\,interactions}, \\
\end{eqnarray*}

which includes the desired physical quantities $\boldsymbol{\lambda }$ (such as magnetic and electric fields, temperature, pressure, etc.), the final state becomes $\mathinner {|{\psi (\boldsymbol{\lambda })}\rangle }=e^{-i H(\boldsymbol{\lambda })t_0}\mathinner {|{\psi _0}\rangle }= \cos {[{\theta (\boldsymbol{\lambda })}/{2}]}\mathinner {|{0}\rangle } +\sin {[{\theta (\boldsymbol{\lambda })}/{2}]}e^{i \varphi (\boldsymbol{\lambda })}\mathinner {|{-1}\rangle }$. Similar to the quasimomentum, these physical quantities $\boldsymbol{\lambda }$ are encoded into the NV center state, allowing us to apply the same measurement protocols for $\boldsymbol{k}$ to measure $\boldsymbol{\lambda }$. Our optimized POVM, capable of achieving the Holevo bound, suggests a significant potential for enhancing measurement precision. Consequently, our multi-parameter estimation methodology, which includes the parameterization and optimization of the POVM and its experimental realization, shows promise for improving the accuracy of measurements in typical NV center applications.

In conclusion, we have demonstrated quantum multi-parameter estimation in a synthetic topological system realized by a highly controllable NV center spin in diamond. By optimizing and implementing POVMs to extract information on two parameters simultaneously, we have achieved the best possible measurement precision characterized by the uncertainty volume and the weighted total variance. We have thus verified the metrological bound set by Berry curvature, and saturated the Holevo bound (namely, the attainable quantum CRB) accessing the limits of quantum multi-parameter estimation. Furthermore, we have experimentally explored the enhanced metrological potential across topological phase transitions. Our work establishes a fundamental connection between quantum metrology and the geometric features of topological band structures. As an example, we elaborate that the developed principles can be extended to a two-band Chern insulator on a torus geometry [64,65,68]. Other connections between metrological performances and the topology of many-body systems can be established through edge properties [69]. Besides, our methodology for multi-parameter estimation, particularly the parameterization and optimization of the POVM and its experimental realization, also holds potential applications for enhancing the precision in measuring physical quantities such as magnetic and electric fields, temperature and pressure in the experiment.

## NOTE ADDED

During the preparation of this manuscript, the authors became aware of the related work by Li *et al.* [29], who experimentally investigated the quantum geometry of quantum multi-parameter sensing.

## Supplementary Material

nwae065_Supplemental_File
