# Supplementary material for: Experimental demonstration of topological bounds in quantum metrology
Source: Natl Sci Rev. 2024 Feb 26;11(10):nwae065. doi: 10.1093/nsr/nwae065 (PMC11409888; doi:10.1093/nsr/nwae065)
Supplement: nwae065_Supplemental_File [file nwae065_supplemental_file.pdf]

Supplementary Materials

Min Yu,[1,2,*] Xiangbei Li,[1,2,*] Yaoming Chu,[1,2,†] Bruno Mera,[3,‡] F. Nur Ünal,[4,§] Pengcheng Yang,[1,2] Yu Liu,[5,2] Nathan Goldman,[6,7,¶] and Jianming Cai[1,2,8,**]

[1]*School of Physics, Hubei Key Laboratory of Gravitation and Quantum Physics, Institute for Quantum Science and Engineering, Huazhong University of Science and Technology, Wuhan 430074, China*
[2]*International Joint Laboratory on Quantum Sensing and Quantum Metrology, Huazhong University of Science and Technology, Wuhan 430074, China*
[3]*Advanced Institute for Materials Research (WPI-AIMR), Tohoku University, Sendai 980-8577, Japan*
[4]*TCM Group, Cavendish Laboratory, University of Cambridge, JJ Thomson Avenue, Cambridge CB3 0HE, United Kingdom*
[5]*Institut für Theoretische Physik and IQST, Albert-Einstein Allee 11, Universität Ulm, Ulm D-89081 Germany*
[6]*Center for Nonlinear Phenomena and Complex Systems, Université Libre de Bruxelles, CP 231, Campus Plaine, B-1050 Brussels, Belgium*
[7]*Laboratoire Kastler Brossel, Collège de France, CNRS, ENS-Université PSL, Sorbonne Université, 11 Place Marcelin Berthelot, 75005 Paris, France*
[8]*Shanghai Key Laboratory of Magnetic Resonance, East China Normal University, Shanghai 200062, China*

A. Bounds for quantum multi-parameter estimation

The quantum Cramér-Rao bound (QCRB) sets a fundamental limit on the accuracy of unbiased quantum parameter estimation, relating the uncertainty in determining parameters to the inverse of the quantum Fisher information (QFI). The QCRB is generally achievable in the case of single parameter estimation (see e.g. Ref. [1]). However, its multi-parameter version is not always attainable due to the incompatibility between optimal measurement operators for different parameters, where the QFI is generalized to QFI matrix (QFIM) [2].

Quantum SLD Cramér-Rao bound

The QCRB states that the measurement uncertainty of the parameters $\boldsymbol{\lambda} = (\lambda_1, \cdots, \lambda_d)$ in dimension d, as characterized by the covariance matrix of the unbiased estimator [2] $\Sigma(\hat{\boldsymbol{\lambda}}) = [\langle \delta\hat{\lambda}_i \delta\hat{\lambda}_j \rangle]_{1\leq i,j\leq d}$ is bounded by the QFIM, $\mathcal{F}_Q(\boldsymbol{\lambda})$, which is related to the quantum metric, $g(\boldsymbol{\lambda}) = [g_{ij}(\boldsymbol{\lambda})]_{1\leq i,j\leq d}$, through the inequality

$$\Sigma(\hat{\boldsymbol{\lambda}}) \geqslant \frac{1}{N}\mathcal{F}_Q^{-1}(\boldsymbol{\lambda}) = \frac{1}{4N} g^{-1}(\boldsymbol{\lambda}), \tag{S.1}$$

where N is the number of measurement repetitions and note that the last equal sign of the above equation holds for the pure state. In the following, we will refer to this bound as the quantum SLD-CRB, due to the fact that the QFI matrix is defined via the symmetric logarithmic derivative (SLD) operator. To be precise, the symmetric logarithmic derivative of a smooth family of density matrices $\rho_{\boldsymbol{\lambda}}$ is the matrix valued one-form, $L = \sum_i L_i d\lambda_i$ defined by

$$d\rho = \frac{1}{2}\left(L\rho + \rho L\right), \tag{S.2}$$

where $d = \sum_i d\lambda_i \frac{\partial}{\partial \lambda_i}$ is the exterior derivative. The quantum Fisher metric is then given by

$$[\mathcal{F}_Q(\boldsymbol{\lambda})]_{ij} = \frac{1}{2}\operatorname{Tr}\left(\rho_{\boldsymbol{\lambda}}\{L_i, L_j\}\right), \; i,j \in \{1,\ldots,d\}, \tag{S.3}$$

where $\{\cdot,\cdot\}$ is the matrix anti-commutator.

In order to quantify the overall performance of quantum multi-parameter estimation, it is convenient to recast the covariance matrix $\Sigma(\hat{\boldsymbol{\lambda}})$ into scalar characteristic functions. One representative scalar characteristic function is given by the generalized variance, i.e. $\det(\Sigma(\hat{\boldsymbol{\lambda}}))$. Based on Eq. (S.1), the bound for the generalized variance is given by

$$\sqrt{\det\left(\Sigma(\hat{\boldsymbol{\lambda}})\right)} \geqslant N^{-d/2}\sqrt{\det\left(\mathcal{F}_Q^{-1}(\boldsymbol{\lambda})\right)} = (4N)^{-d/2}\frac{1}{\sqrt{\det\left(g(\boldsymbol{\lambda})\right)}}. \tag{S.4}$$

Another typical scalar characteristic function corresponds to the weighted total variance, which is bounded by

$$\operatorname{Tr}\left(W\Sigma(\hat{\boldsymbol{\lambda}})\right) \geqslant \frac{1}{N}C^S(\boldsymbol{\lambda}, W) \equiv \frac{1}{N}\operatorname{Tr}\left(W\mathcal{F}_Q^{-1}(\boldsymbol{\lambda})\right) = \frac{1}{4N}\operatorname{Tr}\left(W g^{-1}(\boldsymbol{\lambda})\right), \tag{S.5}$$

where W is a real symmetric matrix. We remark that the attainability of the quantum SLD-CRB is determined by whether it is possible to find a set of POVM $\{M_m\}$ for which the corresponding probabilities $p_m = \mathrm{Tr}(\rho_{\boldsymbol{\lambda}} M_m)$ yield the Fisher information matrix $F_C = \mathcal{F}_Q$, where $[F_C(\boldsymbol{\lambda})]_{ij} \equiv \sum_m p_m(\boldsymbol{\lambda})[\partial \ln p_m(\boldsymbol{\lambda})/\partial\lambda_i][\partial \ln p_m(\boldsymbol{\lambda})/\partial\lambda_j]$. In the single parameter case, such a set of POVM can always be constructed based on the SLD operator. However, the question is much more complex for the quantum multi-parameter estimation. If all the SLDs corresponding to different parameters commute with each other, one may saturate the quantum SLD-CRB by performing a joint measurement of the SLDs. But if the SLDs do not commute, POVMs that are optimal for different parameters are usually incompatible. This observation has led to the increasingly intensive investigation of more effective bounds for quantum multi-parameter estimation.

Berry-curvature bound for generic Dirac systems

Quantum geometry has emerged as a central and ubiquitous concept in quantum sciences, with direct consequences on quantum metrology and many-body quantum physics. The Fubini-Study metric introduces a notion of distance between quantum states defined over the parameter space, and the Berry curvature plays a crucial role in capturing Berry phase effects and topological band structures. The work in Ref. [3] establishes a general and exact relation between these two representative geometric quantities for generic Dirac Hamiltonians as,

$$\frac{i^n}{(2\pi)^n n!}\frac{1}{2^n}\sum_{i_1,j_1,\ldots,i_n,j_n=1}^{2n} \mathrm{Tr}\left(\Omega_{i_1 j_1}\ldots\Omega_{i_n j_n}\right)\varepsilon^{i_1 j_1\ldots i_n j_n} = \mathrm{sgn}(d\vec{n})(-1)^n \frac{(2n)!}{2^{n(n-1)+1} n! \pi^n}\sqrt{\det(g)}, \tag{S.6}$$

where Ω is the Berry curvature, $n = \lfloor D/2 \rfloor$ and D is the number of generators of the complex Clifford algebra. This relationship establishes a general topological bound for the generalized variance in Eq.(S.4),

$$\sqrt{\det(\Sigma(\hat{\boldsymbol{k}}))} > \frac{(2n)!}{N^n 2^{n^2-n+1}} \frac{1}{\left|\sum_{i_1,j_1,\ldots,i_n,j_n=1}^{2n} \mathrm{Tr}\left(\Omega_{i_1 j_1}\ldots\Omega_{i_n j_n}\right)\varepsilon^{i_1 j_1\ldots i_n j_n}\right|}, \tag{S.7}$$

where the momentum parameter $\boldsymbol{k}$ is defined over a d-dimensional Brillouin zone, $\mathbb{T}^d$, with $d = 2n$. As an example, we consider the two-dimensional case where the above inequality reduces to [i.e., Eq.(5) in the main text]

$$\sqrt{\det(\Sigma(\hat{\boldsymbol{k}}))} > \frac{1}{2N}\frac{1}{|\Omega_{12}(\boldsymbol{k})|}. \tag{S.8}$$

Such a topological bound given by the Berry curvature for quantum multi-parameter estimation can be interpreted as a constraint on the momentum estimation by an effective magnetic field in $\boldsymbol{k}$-space. In the main text (see Fig.2), we verify this Berry curvature bound in Eq.(S.8) by experimentally determining the generalized variance $\det(\Sigma(\hat{\boldsymbol{k}}))$ and the quantum geometric tensor (i.e. the quantum metric g and the Berry curvature Ω) separately.

In the following, we give a physical explanation for the Berry curvature's connection to the uncertainty volume in quantum metrology. It is well known that when a quantum system is adiabatically transported around a closed loop in the parameter space, it acquires a geometric phase——the so-called Berry phase. The Berry phase is the integral of the relative phase between neighboring states on the loop [4]. Essentially, Berry curvature is the "flux density" of Berry phase, which measures how the phase of the wavefunction twists as the parameters are varied. *A large Berry curvature means that even a small change in the parameters correlates with a significant change in the wave function, as it is crucial for achieving a larger geometric phase.* Therefore, *intuitively, the Berry curvature can also be viewed as an indicator of how sensitive the system's wave function is to changes in parameters*, akin to the quantum metric. Mathematically, this intuition can be rigorously proved that in general $g_{\mu\mu}g_{\nu\nu} - g_{\mu\nu}^2 \geq \Omega_{\mu\nu}^2/4$ (which is $\det(g) \geq |\Omega_{12}|^2/4$ for two-dimensional parameter space) , see Ref. [3, 5] and references therein. $g_{\mu\mu}g_{\nu\nu} - g_{\mu\nu}^2$ is related to the area element in the two-dimensional surface extended by parameter (μ, ν) within the overall higher-dimensional parameter space. So, a high Berry curvature indicates a large area element $\sqrt{g_{\mu\mu}g_{\nu\nu} - g_{\mu\nu}^2}d\mu d\nu$ at this point, roughly implying greater quantum distances along the parameters μ and ν. This signifies that the state is highly sensitive to changes in both parameters. Consequently, when estimating parameters (μ, ν), their associated uncertainty volume is reduced. Therefore, Berry curvature is directly linked to the magnitude of the uncertainty volume. Furthermore, for generic Dirac Hamiltonians considered here, the exact relationship between the Berry curvature and the square root of determination of quantum metric (for two-dimensional case is $\det(g) = |\Omega_{12}|^2/4$) further underscores this point. It suggests that Berry curvature directly sets a lower bound to the measurement uncertainty volume, linking these two concepts in a fundamental way.

Holevo Cramér-Rao bound

The Holevo Cramér-Rao bound (HCRB), which is referred to as the Holevo bound or the attainable quantum CRB in the main text, is the asymptotically tight bound for general multi-copy estimation models. Given any measurement M_m and an unbiased estimator $\hat{\boldsymbol{\lambda}}$ for the parameter $\boldsymbol{\lambda}$, we define a vector of Hermitian matrices $\mathbf{X} = [X_1, \ldots, X_d]^{\mathrm{T}}$ as [6]

$$\mathbf{X} := \sum_m \left(\hat{\boldsymbol{\lambda}}(m) - \boldsymbol{\lambda}\right) M_m, \tag{S.9}$$

which satisfy the following conditions

$$\mathrm{Tr}\left(\rho_{\boldsymbol{\lambda}}\mathbf{X}\right) = 0, \quad \mathrm{Tr}\left(\boldsymbol{\nabla}\rho_{\boldsymbol{\lambda}}\mathbf{X}^{\mathrm{T}}\right) = \mathcal{I}. \tag{S.10}$$

The Holevo bound is defined by [6]

$$\mathrm{Tr}(W\Sigma(\boldsymbol{\lambda})) \geqslant \frac{1}{N}C^H(\boldsymbol{\lambda}, W) \equiv \frac{1}{N}\min_{\mathbf{X},V}\left(\mathrm{tr}(WV) \mid V \geqslant Z[\mathbf{X}], \quad \mathrm{Tr}\left(\nabla\rho_{\boldsymbol{\lambda}}\mathbf{X}^{\mathrm{T}}\right) = \mathcal{I}\right) \tag{S.11}$$

where V is a $d \times d$ real matrix and $Z[\mathbf{X}] = \mathrm{Tr}\left(\rho_{\boldsymbol{\lambda}}\mathbf{X}\mathbf{X}^{\mathrm{T}}\right)$ is a $d \times d$ complex matrix. In our experiments, we saturate the Holevo bound by performing optimal POVM that minimizes the scalar function $\mathrm{Tr}(WF_C^{-1})$, see Fig.3 in the main text.

The attainable multi-parameter HCRB and the SLD-CRB satisfy the following relationship [7]

$$\begin{aligned} C^S(\boldsymbol{\lambda}, W) &\leq C^H(\boldsymbol{\lambda}, W) \\ &\leq C^S(\boldsymbol{\lambda}, W) + \left\|\sqrt{W}\boldsymbol{\mathcal{F}}_Q^{-1}\boldsymbol{D}\boldsymbol{\mathcal{F}}_Q^{-1}\sqrt{W}\right\|_1 \\ &\leq (1+R)C^S(\boldsymbol{\lambda}, W) \\ &\leq 2C^S(\boldsymbol{\lambda}, W) \end{aligned} \tag{S.12}$$

where $\|A\|_1 = \mathrm{Tr}[\sqrt{A^\dagger A}]$ and $\boldsymbol{D}$ is the (asymptotic) incompatibility matrix (i.e. mean Uhlmann curvature) with element $D_{ij} = -i\,\mathrm{Tr}\left[\rho_{\boldsymbol{\lambda}}\left[L_i, L_j\right]\right]/4$ (for pure states this reduces to the Berry curvature Ω). The parameter $R = \left\|i2\mathcal{F}_Q^{-1}\Omega\right\|_\infty \in [0, 1]$ characterizes the attainability of the scalar SLD-CRB [7–9], where $\|A\|_\infty$ denotes the largest eigenvalue of A. The ratio C^H/C^S quantifies the discrepancy between the HCRB and the SLD-CRB which can be written as

$$1 \leq C^H/C^S \leq 1 + R \leq 2. \tag{S.13}$$

Our experiment provides the lower bound of R by direct metrological determination of $C^H(\boldsymbol{k}, W)$ and geometric measurement of $C^S(\boldsymbol{k}, W)$, see Fig.3 (c-d) in the main text.

B. Parameterization and optimization of POVM

Parameterization of POVM

Given a measurement observable, one can use the maximum likelihood estimator to extract the information on the unknown parameters. The corresponding covariance matrix Σ is given by the inverse of the Fisher information matrix, i.e. F_C^{-1}. The performance of quantum parameter estimation is generally dependent on the measurement observable. In order to achieve the best metrological performance, we proceed to find the optimal POVM by minimizing the values of $\sqrt{\det F_C^{-1}}$ and $\mathrm{Tr}(WF_C^{-1})$.

We remark that in order to estimate n parameters, the POVM usually needs to have at least $(n+1)$ elements, e.g. 3-element POVM may allow us to estimate two parameters simultaneously. Moreover, we find that the Holevo bound actually can be attained by optimizing the 3-element rank-1 POVM. Therefore, we start by considering a set of 3-element rank-1 POVM,

$$\Pi_i = \left|e_i\right\rangle\left\langle e_i\right|, \quad i = 1, 2, 3, \tag{S.14}$$

which satisfies the following normalization condition

$$\sum_{i=1}^{3}\Pi_i = \mathbb{1}. \tag{S.15}$$

The state vector $|e_i\rangle$ can be parameterized as

$$|e_i\rangle = r_i\left(\cos\frac{\theta_i}{2}|0\rangle + \sin\frac{\theta_i}{2}e^{i\varphi_i}|-1\rangle\right), \tag{S.16}$$

where $|0\rangle$ and $|-1\rangle$ denote the spin sublevel $m_s = 0$ and $m_s = -1$ of the NV center respectively. Based on Eq. (S.16), nine parameters are needed to determine a set of 3-element rank-1 POVM. The normalization condition in Eq. (S.15) leads to

$$\begin{aligned} &r_1^2 + r_2^2 + r_3^2 = 2,\\ &r_1^2\cos\theta_1 + r_2^2\cos\theta_2 + r_3^2\cos\theta_3 = 0,\\ &r_1^2\sin\theta_1\cos\varphi_1 + r_2^2\sin\theta_2\cos\varphi_2 + r_3^2\sin\theta_3\cos\varphi_3 = 0,\\ &r_1^2\sin\theta_1\sin\varphi_1 + r_2^2\sin\theta_2\sin\varphi_2 + r_3^2\sin\theta_3\sin\varphi_3 = 0. \end{aligned} \tag{S.17}$$

which further reduces the number of free parameters in Eq. (S.16) to five. In addition, the last three equations of Eq. (S.16) tells that the three vectors defined by $\vec{n}_i = (\sin\theta_i\cos\varphi_i, \sin\theta_i\sin\varphi_i, \cos\theta_i)$ $(i = 1, 2, 3)$ are in the same plane, i.e. $r_1^2\vec{n}_1 + r_2^2\vec{n}_2 + r_3^2\vec{n}_3 = 0$. Next, we first introduce the following three state vectors in the $x - y$ plane,

$$\begin{aligned} |e_{10}\rangle &= \frac{r_1}{\sqrt{2}}\left(|0\rangle + |-1\rangle\right),\\ |e_{20}\rangle &= \frac{r_2}{\sqrt{2}}\left(|0\rangle + e^{-i\vartheta}|-1\rangle\right),\\ |e_{30}\rangle &= \frac{r_3}{\sqrt{2}}\left(|0\rangle + e^{i\phi}|-1\rangle\right), \end{aligned} \tag{S.18}$$

with

$$\vartheta = \arccos\left[(r_3^4 - r_1^4 - r_2^4)/(2r_1^2r_2^2)\right], \tag{S.19}$$

$$\phi = \arccos\left[(r_2^4 - r_1^4 - r_3^4)/(2r_1^2r_3^2)\right]. \tag{S.20}$$

Only two parameters of $\{r_1, r_2, r_3\}$ are independent, without loss of generality, we set $r_1 \leqslant r_2 \leqslant r_3$ and $r_3 = (2 - r_1^2 - r_2^2)^{1/2}$, which satisfy the following conditions

$$\begin{aligned} 0 \leqslant &r_1 \leqslant \sqrt{\frac{2}{3}},\\ \max\left(r_1, \sqrt{1 - r_1^2}\right) \leqslant &r_2 \leqslant \sqrt{1 - \frac{r_1^2}{2}}. \end{aligned} \tag{S.21}$$

The general state vectors $|e_i\rangle$ in Eq.(S.16) can be further obtained by first rotating $\{|e_{i0}\rangle\}$ to make the norm vector of the plane where $\{|e_i\rangle\}$ are located to a specific direction $\hat{n} = (\sin\alpha\cos\beta, \sin\alpha\sin\beta, \cos\alpha)$ and then rotating them around the $\hat{n}$ direction by an angle γ, namely

$$|e_i(r_1, r_2, \alpha, \beta, \gamma)\rangle = U(\alpha, \beta, \gamma)\,|e_{i0}(r_1, r_2)\rangle, \quad i = 1, 2, 3, \tag{S.22}$$

where

$$U(\alpha, \beta, \gamma) = \exp(\frac{-i\hat{n}\cdot\vec{\sigma}\gamma}{2})\exp(\frac{-i\sigma_z\beta}{2})\exp(\frac{-i\sigma_y\alpha}{2}) \quad \text{with} \quad \hat{n} = (\sin\alpha\cos\beta, \sin\alpha\sin\beta, \cos\alpha). \tag{S.23}$$

Such an parameterization allows us to optimize the POVM $\{\Pi_i\}_{i=1}^3$ with the required target characteristic function over the parameter space of $(r_1, r_2, \alpha, \beta, \gamma)$. More specifically, based on the measurement probability of $|\psi(k_1, k_2)\rangle$ in $|e_i\rangle$, i.e.,

$$P_i(k_1, k_2;\, r_1, r_2, \alpha, \beta, \gamma) = |\langle\psi(k_1, k_2)|e_i(r_1, r_2, \alpha, \beta, \gamma)\rangle|^2, \tag{S.24}$$

the corresponding Fisher information matrix is given by

$$F_C(k_1, k_2;\, r_1, r_2, \alpha, \beta, \gamma) = \begin{pmatrix} [F_C]_{k_1k_1} & [F_C]_{k_1k_2}\\ [F_C]_{k_2k_1} & [F_C]_{k_2k_2} \end{pmatrix}, \tag{S.25}$$

with

$$[F_C]_{ij} = \sum_{k=1}^{3} \frac{1}{P_k} \frac{\partial P_k}{\partial \lambda_i} \frac{\partial P_k}{\partial \lambda_j}, \; i,j \in \{k_1, k_2\}. \tag{S.26}$$

We numerically optimize the parameters $\{r_1, r_2, \alpha, \beta, \gamma\}$ to find the minimum of $\sqrt{\det F_C^{-1}}$ and $\mathrm{Tr}(W F_C^{-1})$. The ranges of r_1 and r_2 are shown in Eq. (S.21) and we have $0 \le \alpha \le \pi$, $0 \le \beta \le 2\pi$ and $0 \le \gamma \le 2\pi$, so the search area is manageable. Thus, by optimizing all five parameters, we can efficiently locate both the minimum value of the target function and the corresponding parameters. Furthermore, we implement an alternative method to verify our results. This involves initially fixing the parameters (r_1, r_2) and optimizing α, β, γ, then varying (r_1, r_2) within their ranges to find the final minimum. This secondary approach consistently yielded the same minimum value as the primary method, thereby reinforcing the reliability and robustness of our results. We also note that more efficient methods, such as gradient descent or simulated annealing, could be employed to further improve the optimization efficiency.

Three specific examples of POVM

In the main text, we investigate three different sets of POVM and compare their performance in quantum multi-parameter estimation. The first set of POVM (which denoted as oPOVM) is given by Eq.(S.22) with the parameter values as

$$\begin{aligned} r_1 = r_2 = \sqrt{\frac{2}{3}} \\ \alpha = \theta(k_1, k_2), \beta = \varphi(k_1, k_2), \gamma = \pi. \end{aligned} \tag{S.27}$$

In Fig. S.1(a), we show that $\sqrt{\det F_C^{-1}}$ associated with the oPOVM reaches the minimal value of $\sqrt{\det F_C^{-1}}$ over all 3-element rank-1 POVMs. Besides, we find that the Fisher information matrix of oPOVM is given by

$$F_C = \begin{pmatrix} 1/2 & 0 \\ 0 & \sin^2\theta/2 \end{pmatrix} = \frac{1}{2}\mathcal{F}_{S^2}. \tag{S.28}$$

In Fig. S.1(b),we demonstrate that oPOVM can also saturate the Holevo Cramér-Rao bound for the weight matrix $W_1 = \mathcal{F}_{S^2}$ when estimating θ and φ. Therefore, the oPOVM is optimal for $\mathrm{Tr}(W_1 \Sigma(\hat{k}_1, \hat{k}_2))$ with $W_1 = J^T \mathcal{F}_{S^2} J = \mathcal{F}_{\mathbb{T}^2}$ when estimating $\boldsymbol{k}$, where J is the Jacobian matrix defined in Eq.(S.60). We note that the oPOVM is not optimal for another choice of the weight matrix $W_2 = I$ when estimating θ and φ (which corresponds to the weight matrix $W_2 = J^T J$ when estimating $\boldsymbol{k}$). Nevertheless, we can find another POVM, denoted as oPOVM(W_2), that attains the Holevo Cramér-Rao bound associated with the weight matrix W_2, by optimizing over 3-element rank-1 POVMs, see Fig. S.1(c).

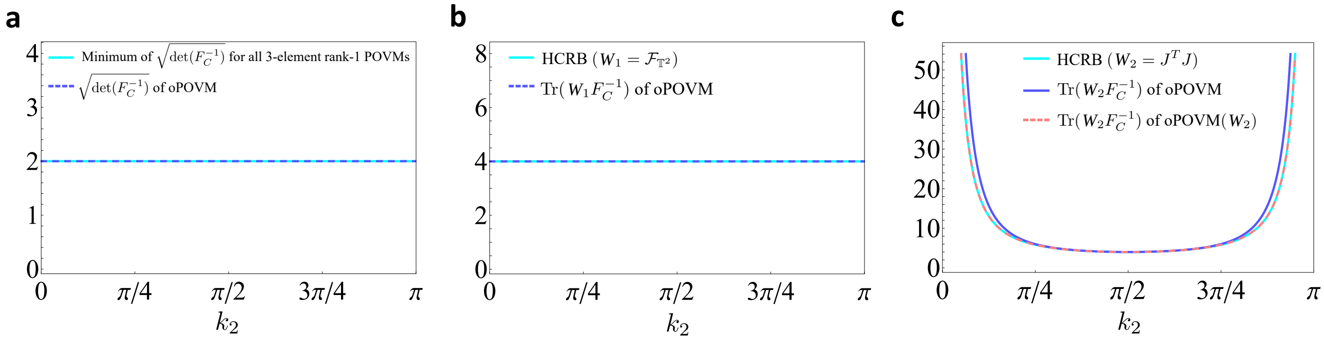

FIG. S.1. (a) $\sqrt{\det F_C^{-1}}$ associated with the oPOVM reaches the minimal value of $\sqrt{\det F_C^{-1}}$ over all 3-element rank-1 POVMs. (b) The oPOVM saturates the Holevo Cramér-Rao bound for the weight matrix $W_1 = \mathcal{F}_{\mathbb{T}^2}$. (c) The oPOVM does not attain the Holevo Cramér-Rao bound for the other choice of the weight matrix $W_2 = J^T J$, while the oPOVM(W_2) that is obtained by optimization saturates the Holevo Cramér-Rao bound.

The second set of POVM that we consider is a symmetric state-independent POVM (denoted as sPOVM below), which is given by Eq.(S.22) with the following parameter values

$$r_1 = r_2 = \sqrt{\frac{2}{3}},\\ \alpha = \pi/2, \beta = \pi/2, \gamma = \pi. \tag{S.29}$$

The corresponding state vectors $|e_i\rangle$ can be written as

$$\begin{aligned} |e_1\rangle &= \sqrt{\frac{2}{3}}\,|0\rangle\,, \\ |e_2\rangle &= -\sqrt{\frac{1}{6}}\,|0\rangle + \sqrt{\frac{1}{2}}\,|-1\rangle\,, \\ |e_3\rangle &= -\sqrt{\frac{1}{6}}\,|0\rangle - \sqrt{\frac{1}{2}}\,|-1\rangle\,. \end{aligned} \tag{S.30}$$

The third set of POVM in the main text is the symmetric, informationally complete POVM (SIC-POVM)[10–12] $\{|e_i\rangle\langle e_i|\}_{i=1}^4$ that has $d^2 = 4$ elements . The SIC-POVM represents the POVM with the fewest elements that can span the space of self-adjoint operators. The corresponding state vectors are given as follows

$$\begin{aligned} |e_1\rangle &= \frac{1}{\sqrt{2}}\,|0\rangle\,, \\ |e_2\rangle &= \frac{1}{\sqrt{6}}\,|0\rangle + \frac{1}{\sqrt{3}}\,|-1\rangle\,, \\ |e_3\rangle &= \frac{1}{\sqrt{6}}\,|0\rangle + e^{i\frac{2\pi}{3}}\frac{1}{\sqrt{3}}\,|-1\rangle\,, \\ |e_4\rangle &= \frac{1}{\sqrt{6}}\,|0\rangle + e^{-i\frac{2\pi}{3}}\frac{1}{\sqrt{3}}\,|-1\rangle\,. \end{aligned} \tag{S.31}$$

C. Experimental realization of POVM

State-dependent fluorescence measurement

In the experiment, the state-dependent fluorescence for the realization of readout is obtained by counting the accumulated photons over many sweeps. For example, we consider to prepare the system in the spin state $|0\rangle$ and collect the signal photons of ν sweeps, where ν is usually set as a very large number. The number n_{0i} of signal photons collected in the ith sweep is a random variable, and we denote its expectation and variance as $\langle n_{0i}\rangle$ and $\sigma^2_{n_{0i}}$. Then the total photons collected in ν sweeps is given by

$$n_0 = \sum_{i=1}^{\nu} n_{0i}, \tag{S.32}$$

which obeys the following normal distribution according to the central limit theorem ($\nu \gg 1$),

$$n_0 \sim \mathcal{N}\left(\nu\langle n_{0i}\rangle, \nu\sigma^2_{n_{0i}}\right) \equiv \mathcal{N}(\langle n_0\rangle, \sigma^2_{n_0}), \tag{S.33}$$

with $\langle n_0\rangle \equiv \nu\langle n_{0i}\rangle$, $\sigma^2_{n_0} \equiv \nu\sigma^2_{n_{0i}}$. We note that similar distributions hold for the signal photons when the system is in state $|-1\rangle$ or $|1\rangle$, namely

$$\begin{aligned} n_{-1} &\sim \mathcal{N}\left(\nu\langle n_{-1i}\rangle, \nu\sigma^2_{n_{-1i}}\right) \equiv \mathcal{N}(\langle n_{-1}\rangle, \sigma^2_{n_{-1}}), \\ n_1 &\sim \mathcal{N}\left(\nu\langle n_{1i}\rangle, \nu\sigma^2_{n_{1i}}\right) \equiv \mathcal{N}(\langle n_1\rangle, \sigma^2_{n_1}). \end{aligned} \tag{S.34}$$

Projective measurement in an extended Hilbert space

In our experiments, the system is encoded in the Hilbert space spanned by $\{|0\rangle, |-1\rangle\}$. We realize the 3-element POVM on the system via the projective measurement in the extended three-level Hilbert space $\{|0\rangle, |-1\rangle, |+1\rangle\}$. First, we choose the projective basis states $|u_i\rangle$ such that

$$(|0\rangle\langle 0| + |-1\rangle\langle -1|)|u_i\rangle = |e_i\rangle . \tag{S.35}$$

For $|e_i\rangle$ in Eq.(3) of the main text, the explicit form of $|u_i\rangle = \langle 0|u_i\rangle|0\rangle + \langle -1|u_i\rangle|-1\rangle + \langle +1|u_i\rangle|+1\rangle$ is given by

$$|u_1\rangle = r_1 \cos\frac{\theta_1}{2}|0\rangle + r_1 \sin\frac{\theta_1}{2}e^{i\varphi_1}|-1\rangle + \sqrt{1-r_1^2}|+1\rangle , \tag{S.36}$$

$$|u_2\rangle = r_2 \cos\frac{\theta_2}{2}|0\rangle + r_1 \sin\frac{\theta_2}{2}e^{i\varphi_2}|-1\rangle - \sqrt{1-r_2^2}e^{i\arg\langle e_1|e_2\rangle}|+1\rangle , \tag{S.37}$$

$$|u_3\rangle = r_3 \cos\frac{\theta_3}{2}|0\rangle + r_3 \sin\frac{\theta_3}{2}e^{i\varphi_3}|-1\rangle - \sqrt{1-r_3^2}e^{i\arg\langle e_1|e_3\rangle}|+1\rangle . \tag{S.38}$$

By such a choice, we have

$$\mathrm{Tr}(\Pi_i\rho_{\boldsymbol{k}}) = \mathrm{Tr}(|e_i\rangle\langle e_i|\rho_{\boldsymbol{k}}) = \langle u_i|\rho_{\boldsymbol{k}}|u_i\rangle \equiv p_i \tag{S.39}$$

In order to realize the projective measurement $\{|u_i\rangle\langle u_i|\}$, we apply a unitary rotation U_1 on the NV center spin, which rotates the system by the following transformation

$$\begin{aligned} U_1|u_1\rangle &= |+1\rangle , \\ U_1|u_2\rangle &= |0\rangle , \\ U_1|u_3\rangle &= |-1\rangle . \end{aligned} \tag{S.40}$$

The subsequent state-dependent fluorescence measurement provides the number of accumulated photons $n_j(1)$. Similarly, we apply another unitary rotation U_2 on the NV center spin, which rotates the system by the following transformation

$$\begin{aligned} U_2|u_1\rangle &= |+1\rangle , \\ U_2|u_2\rangle &= |-1\rangle , \\ U_2|u_3\rangle &= |0\rangle . \end{aligned} \tag{S.41}$$

The corresponding number of accumulated photons is denoted as $n_j(2)$. The unitary rotations are implemented by engineering microwave driving fields on resonance with both transitions ($0 \leftrightarrow -1$ and $0 \leftrightarrow +1$), see Fig.S.2. The corresponding driven Hamiltonians are written as

$$H_d^{(1)} = H_0 + \Omega_1\cos(\omega_1 t + \beta)(|0\rangle\langle -1| + |-1\rangle\langle 0|), \quad t \in [t_1, t_1+\tau_1] \tag{S.42}$$

$$H_d^{(2)} = H_0 + \Omega_2\cos(\omega_2 t + \beta)(|0\rangle\langle +1| + |+1\rangle\langle 0|), \quad t \in [t_2, t_2+\tau_2] \tag{S.43}$$

with $H_0 = \omega_1|-1\rangle\langle -1| + \omega_2|+1\rangle\langle +1|$, where $\omega_{1,2}$ denote the energy gaps between the states $|0\rangle, |-1\rangle$ and $|0\rangle, |+1\rangle$ respectively, and $\tau_{1,2}$ represent the time duration of individual microwave-field pulses. With different values of the phase β, the above driven Hamiltonians are able to achieve arbitrary rotations in the subspaces spanned by $\{|0\rangle, |-1\rangle\}$ and $\{|0\rangle, |+1\rangle\}$ respectively. In order to implement the unitary rotation U_1, we first rotate the projective part of $|u_1\rangle$ in the subspace of $\{|0\rangle, |-1\rangle\}$ to the state $|0\rangle$ by invoking $H_d^{(1)}$. Then, the obtained superposition state of $|0\rangle$ and $|+1\rangle$ is further rotated to the state $|+1\rangle$ through the second driven Hamiltonian $H_d^{(2)}$. In this process, $|u_2\rangle$ and $|u_3\rangle$ are rotated to the subspace of $\{|0\rangle, |-1\rangle\}$. Finally, we again apply $H_d^{(1)}$ to rotate $|u_2\rangle$ and $|u_3\rangle$ to the state $|0\rangle$ and $|-1\rangle$. Hence, we realize a unitary transformation in Eq. (S.40). We note that the other unitary transformation U_2 can be implemented in a similar way.

After applying the unitary transformations U_1 and U_2 [see Eq.(S.40) and Eq.(S.41)], using Eq.(S.33-S.34) and the Lyapunov central limit theorem, the number of signal photons collected in the total ν sweeps will be

$$n_j(1) \sim \mathcal{N}\left(p_1\langle n_1\rangle + p_2\langle n_0\rangle + p_3\langle n_{-1}\rangle,\ p_1\sigma_{n_1}^2 + p_2\sigma_{n_0}^2 + P_3\sigma_{n_{-1}}^2\right), \tag{S.44}$$

$$n_j(2) \sim \mathcal{N}\left(p_1\langle n_1\rangle + p_2\langle n_{-1}\rangle + p_3\langle n_0\rangle,\ p_1\sigma_{n_1}^2 + p_2\sigma_{n_{-1}}^2 + p_3\sigma_{n_0}^2\right). \tag{S.45}$$

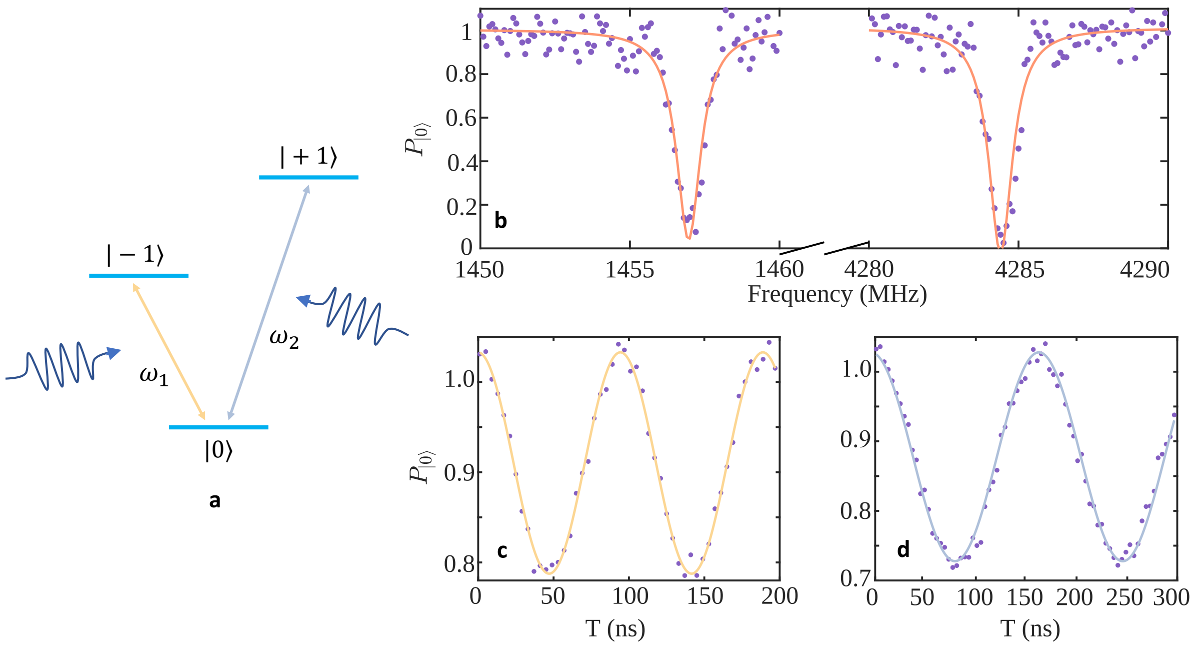

FIG. S.2. (a) The energy level structure of the NV center spin in diamond under an external magnetic field and microwave driving. (b) We measure the energy gap $\omega_{1,2}$ through pulsed optically detected magnetic resonance (Pulsed-ODMR). (c-d) Rabi oscillations between the states $|0\rangle$, $|-1\rangle$ and $|0\rangle$, $|+1\rangle$, respectively.

Based on the obtained photon number $n_j(1)$ and $n_j(2)$, we can get the following probability associated with the j-th measurement as

$$\begin{aligned} p_{1j} &= \frac{\langle n_0\rangle + \langle n_{-1}\rangle - n_j(1) - n_j(2)}{\langle n_0\rangle + \langle n_{-1}\rangle - 2\langle n_1\rangle}, \\ p_{2j} &= \frac{(\langle n_0\rangle - \langle n_1\rangle)\, n_j(1) + (\langle n_1\rangle - \langle n_{-1}\rangle) n_j(2) - (\langle n_0\rangle - \langle n_{-1}\rangle)\langle n_1\rangle}{(\langle n_0\rangle - \langle n_{-1}\rangle)(\langle n_0\rangle + \langle n_{-1}\rangle - 2\langle n_1\rangle)}, \\ p_{3j} &= 1 - p_{1j} - p_{2j}, \end{aligned} \tag{S.46}$$

which satisfy the following equations

$$\begin{aligned} &p_{1j} + p_{2j} + p_{3j} = 1 \\ &p_{1j}\langle n_1\rangle + p_{2j}\langle n_0\rangle + p_{3j}\langle n_{-1}\rangle = n_j(1) \\ &p_{1j}\langle n_1\rangle + p_{2j}\langle n_{-1}\rangle + p_{3j}\langle n_0\rangle = n_j(2). \end{aligned} \tag{S.47}$$

Note that for two independent random variables $\xi \sim \mathcal{N}(\mu_1, \sigma_1^2)$ and $\eta \sim \mathcal{N}(\mu_2, \sigma_2^2)$, $a\xi + b\eta \sim \mathcal{N}(a\mu_1 + b\mu_2, a^2\sigma_1^2 + b^2\sigma_2^2)$ holds. Hence, p_{1j} and p_{2j} obey normal distributions as well, namely

$$\begin{aligned} p_{1j} &\sim \mathcal{N}\left(p_1,\, \tilde{\sigma}_1^2\right), \\ p_{2j} &\sim \mathcal{N}\left(p_2,\, \tilde{\sigma}_2^2\right), \\ p_{3j} &\sim \mathcal{N}\left(p_3,\, \tilde{\sigma}_3^2\right), \end{aligned} \tag{S.48}$$

with the variance $\tilde{\sigma}_i^2$ $(i = 1, 2, 3)$ given by

$$\begin{aligned} \tilde{\sigma}_1^2 &= \frac{2p_1\sigma_{n_1}^2 + (p_2 + p_3)(\sigma_{n_0}^2 + \sigma_{n_{-1}}^2)}{(\langle n_0\rangle + \langle n_{-1}\rangle - 2\langle n_1\rangle)^2} \\ \tilde{\sigma}_2^2 &= \frac{(\langle n_0\rangle - \langle n_1\rangle)^2 (p_1\sigma_{n_1}^2 + p_2\sigma_{n_0}^2 + p_3\sigma_{n_{-1}}^2) + (\langle n_1\rangle - \langle n_{-1}\rangle)^2 (p_1\sigma_{n_1}^2 + p_2\sigma_{n_{-1}}^2 + p_3\sigma_{n_0}^2)}{(\langle n_0\rangle - \langle n_{-1}\rangle)^2(\langle n_0\rangle + \langle n_{-1}\rangle - 2\langle n_1\rangle)^2}, \\ \tilde{\sigma}_3^2 &= \frac{(\langle n_1\rangle - \langle n_{-1}\rangle)^2 (p_1\sigma_{n_1}^2 + p_2\sigma_{n_0}^2 + p_3\sigma_{n_{-1}}^2) + (\langle n_0\rangle - \langle n_1\rangle)^2 (p_1\sigma_{n_1}^2 + p_2\sigma_{n_{-1}}^2 + p_3\sigma_{n_0}^2)}{(\langle n_0\rangle - \langle n_{-1}\rangle)^2(\langle n_0\rangle + \langle n_{-1}\rangle - 2\langle n_1\rangle)^2}. \end{aligned} \tag{S.49}$$

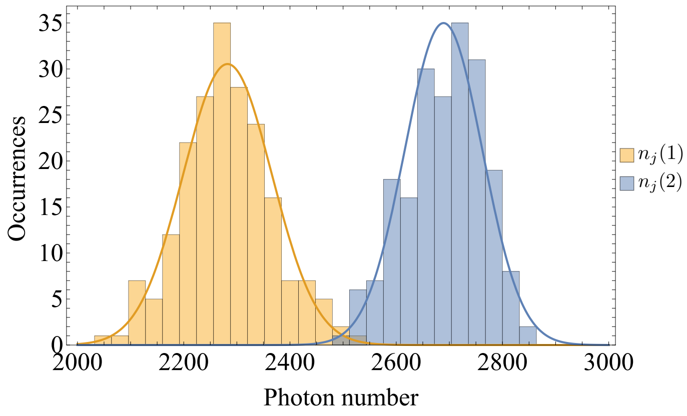

FIG. S.3. An example of the histogram of occurrences of $n_j(1)$ and $n_j(2)$ in the experiment.

We remark that these variance are inversely proportional to the number of sweeps ν, i.e. $\tilde{\sigma}_1^2 \propto \nu^{-1}$, $\tilde{\sigma}_2^2 \propto \nu^{-1}$, which indicates that the fluctuation of p_{1j} and p_{2j} can be reduced by increasing the number of sweeps in the jth-experiment. Here, we assume that $\tilde{\sigma}_i^2$ is small enough to ensure that $p_{1j} > 0$ and $p_{2j} > 0$. Therefore, according to Eq.(S.39), we can obtain the measurement probabilities for the POVM $\{\Pi = |e_i\rangle\langle e_i|\}_{i=1}^3$, namely

$$\mathrm{Tr}\left(\Pi_i \rho_{\boldsymbol{k}}\right) = \langle u_i| \rho_{\boldsymbol{k}} |u_i\rangle = p_i = \langle p_{ij}\rangle. \tag{S.50}$$

We can then assign $x_j \in \{1, 0, -1\}$ as the measurement outcome of the j-th experimental according to the obtained probabilities p_{1j}, p_{2j} and p_{3j} respectively [1].

D. Experimental procedure to extract the covariance matrix

The full covariance matrix $\Sigma(\hat{\boldsymbol{k}})$ contains not only the measurement uncertainties for the j-th parameter $\langle \delta\hat{\boldsymbol{k}}_j^{\,2}\rangle$, but also the cross elements $\langle \delta\hat{\boldsymbol{k}}_i \delta\hat{\boldsymbol{k}}_j\rangle$. In this section, we will present details on how to experimentally extract the covariance matrix $\Sigma(\hat{k}_1, \hat{k}_2)$ and thereby directly determine the performance of quantum multi-parameter estimation protocols.

Maximum likelihood estimator

We consider the general POVM $\{\Pi_i\}_{i=1}^m$ with m elements. The measurement outcome corresponding to the i-th measurement Π_i is denoted as a_i, with the probability $p_i(\boldsymbol{k}) = \mathrm{Tr}[\Pi_i \rho_{\boldsymbol{k}}]$. By performing N repetitive measurements, we obtain a series of measurement outcomes as $\vec{x} = (x_1,\ x_2, \cdots,\ x_j, \cdots,\ x_N)$, where $x_j \in (a_1, \cdots, a_m)$ associated with the probability $P_j(x_j, \boldsymbol{k}) \in \{p_1(\boldsymbol{k}), \cdots, p_m(\boldsymbol{k})\}$. Next, we present the detailed steps to construct the maximum likelihood estimator (MLE) $\hat{\boldsymbol{k}}$ based on $\vec{x}$. The log-likelihood function is

$$\ell(\boldsymbol{k}; \vec{x}) = \sum_{j=1}^{N} \ln P_j(x_j, \boldsymbol{k}). \tag{S.51}$$

The MLE $\hat{\boldsymbol{k}}$ maximizes $\ell(\boldsymbol{k}; \vec{x})$ and thus satisfies the likelihood equations as $\partial\ell/\partial k_1|_{\boldsymbol{k}=\hat{\boldsymbol{k}}} = 0,\ \ \partial\ell/\partial k_2|_{\boldsymbol{k}=\hat{\boldsymbol{k}}} = 0$, namely

$$\begin{aligned} \sum_{j=1}^{N} \frac{1}{P_j(x_j, \hat{\boldsymbol{k}})} \frac{\partial P_j(x_j, \hat{\boldsymbol{k}})}{\partial \hat{k}_1} &= \sum_{i=1}^{m} \frac{n_i}{p_i(\hat{\boldsymbol{k}})} \frac{\partial p_i(\hat{\boldsymbol{k}})}{\partial \hat{k}_1} = 0, \\ \sum_{j=1}^{N} \frac{1}{P_j(x_j, \hat{\boldsymbol{k}})} \frac{\partial P_j(x_j, \hat{\boldsymbol{k}})}{\partial \hat{k}_2} &= \sum_{i=1}^{m} \frac{n_i}{p_i(\hat{\boldsymbol{k}})} \frac{\partial p_i(\hat{\boldsymbol{k}})}{\partial \hat{k}_2} = 0, \end{aligned} \tag{S.52}$$

where n_i is the number of occurrences of a_i in $\vec{x}$. We construct the probability estimator of $p_i(\boldsymbol{k})$ as follows

$$\hat{p}_i(\vec{x}) = \frac{1}{N}\sum_{j=1}^{N}\delta_{a_i,x_j} \equiv \frac{1}{N}\sum_{j=1}^{N} y_i^{(j)} = \frac{n_i}{N}. \tag{S.53}$$

According to Eq. (S.52), we can get

$$\begin{aligned}\sum_{i=1}^{m}\frac{\hat{p}_i(\vec{x})}{p_i(\hat{\boldsymbol{k}})}\frac{\partial p_i(\hat{\boldsymbol{k}})}{\partial \hat{k}_1} = 0,\\ \sum_{i=1}^{m}\frac{\hat{p}_i(\vec{x})}{p_i(\hat{\boldsymbol{k}})}\frac{\partial p_i(\hat{\boldsymbol{k}})}{\partial \hat{k}_2} = 0.\end{aligned} \tag{S.54}$$

Using the normalization condition $\sum_i^m \hat{p}_i = 1$, the MLE $\hat{\boldsymbol{k}}$ as functions of $\{\hat{p}_i(\vec{x})\}_{i=1}^{m-1}$ can be solved based on Eq. (S.54),

$$\hat{\boldsymbol{k}}(\vec{x}) = \hat{\boldsymbol{k}}\left(\hat{p}_1(\vec{x}), \hat{p}_2(\vec{x}), \cdots, \hat{p}_{m-1}(\vec{x})\right). \tag{S.55}$$

It is generally a challenging task to get the analytical expression of $\hat{\boldsymbol{k}}\left(\hat{p}_1, \hat{p}_2, \cdots, \hat{p}_{m-1}\right)$, thus one usually needs to solve Eq. (S.54) numerically in order to get the maximum likelihood estimator.

We note that when the number of POVM elements m is equal to to the number of parameters plus one, the maximum likelihood estimator have a simple form. In the experiment, we use the set of 3-element POVM. In such a scenario, we have

$$\begin{aligned}p_1 &= p_1(k_1, k_2)\\ p_2 &= p_2(k_1, k_2)\\ p_3 &= p_3(k_1, k_2) = 1 - p_1(k_1, k_2) - p_2(k_1, k_2).\end{aligned} \tag{S.56}$$

Under the assumption that $(p_1,\ p_2)$ and $(k_1,\ k_2)$ are one-to-one mapping, we can solve k_1 and k_2 as functions of p_1 and p_2 as

$$\begin{aligned}k_1 &= k_1(p_1, p_2),\\ k_2 &= k_2(p_1, p_2).\end{aligned} \tag{S.57}$$

Then, one can prove that the following estimators

$$\begin{aligned}\hat{k}_1 &= k_1(\hat{p}_1, \hat{p}_2),\\ \hat{k}_2 &= k_2(\hat{p}_1, \hat{p}_2),\end{aligned} \tag{S.58}$$

satisfy the likelihood equations and thus yield the MLE $\hat{\boldsymbol{k}}$.

Extracting the covariance matrix from measurements

By exploiting the one-to-one map between $\boldsymbol{k} = (k_1, k_2) \leftrightarrow \boldsymbol{n_k} = (\theta_{\boldsymbol{k}}, \varphi_{\boldsymbol{k}})$ in a suitable parameter range, the covariance matrix of the MLE in Eq. (S.58) can be expressed as

$$\begin{aligned}\Sigma(\hat{k}_1, \hat{k}_2) &= \left[\frac{\partial(\hat{k}_1, \hat{k}_2)}{\partial(\hat{p}_1, \hat{p}_2)}\right]\Sigma(\hat{p}_1, \hat{p}_2)\left[\frac{\partial(\hat{k}_1, \hat{k}_2)}{\partial(\hat{p}_1, \hat{p}_2)}\right]^T\\ &= J^{-1}\left[\frac{\partial(\hat{\theta}, \hat{\varphi})}{\partial(\hat{p}_1, \hat{p}_2)}\right]\Sigma(\hat{p}_1, \hat{p}_2)\left[\frac{\partial(\hat{\theta}, \hat{\varphi})}{\partial(\hat{p}_1, \hat{p}_2)}\right]^T (J^{-1})^T\end{aligned} \tag{S.59}$$

where J is the Jacobian matrix written as follows

$$J = \frac{\partial(\theta, \varphi)}{\partial(k_1, k_2)}. \tag{S.60}$$

Note that for the MLE of 3-element POVM, $\partial(\hat{\theta}, \hat{\varphi})/\partial(\hat{p}_1, \hat{p}_2)$ and $\partial(\theta, \varphi)/\partial(p_1, p_2)$ have the same functional form, therefore we can obtain $\partial(\hat{\theta}, \hat{\varphi})/\partial(\hat{p}_1, \hat{p}_2)$ from the slope of the signals, see Fig. S.4.

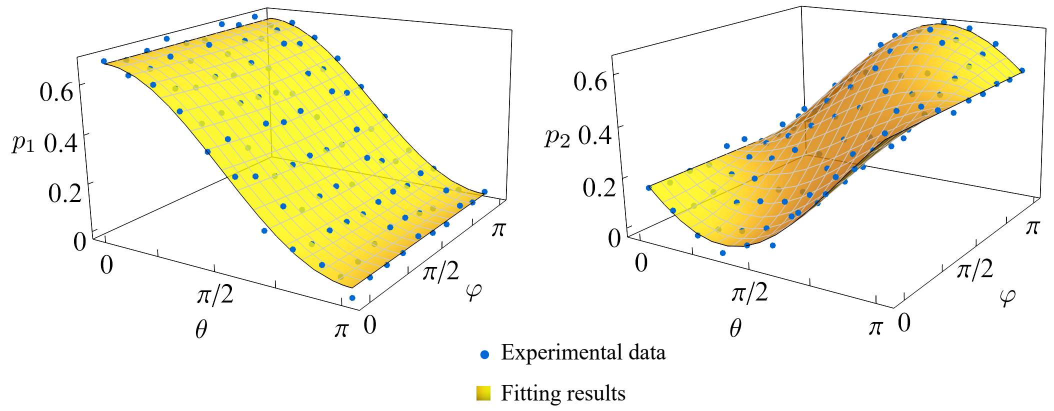

FIG. S.4. Examples of the experimental measurement of $\partial(\hat{\theta},\hat{\varphi})/\partial(\hat{p}_1,\hat{p}_2)$. From Eq. (S.46) and (S.48), we can estimate $p_1(\theta,\varphi)$ and $p_2(\theta,\varphi)$ in the experiment. By measuring p_1, p_2 at different (θ,φ), we derive the fitting functions $p_1(\theta,\varphi)$ and $p_2(\theta,\varphi)$. Based on the fitting results, we can obtain the derivative components in the Jacobian matrix. (a) Measurement of $\partial p_1/\partial\theta$ and $\partial p_1/\partial\varphi$ by fitting $p_1(\theta,\varphi)$. (b) Measurement of $\partial p_2/\partial\theta$ and $\partial p_2/\partial\varphi$ by fitting $p_2(\theta,\varphi)$.

Note that the definition for $\{\hat{p}_1,\hat{p}_2\}$ in Eq.(S.53) can be re-expressed as

$$\hat{p}_i(\vec{x}) = \frac{1}{N}\sum_{j=1}^{N} y_i^{(j)}, \tag{S.61}$$

with $y_i^{(j)} \equiv \delta_{a_i,x_j}$, i.e.

$$\begin{aligned} y_1^{(j)} &= \delta_{a_1,x_j} = \delta_{1,x_j} = \frac{x_j+|x_j|}{2}, \\ y_2^{(j)} &= \delta_{a_2,x_j} = \delta_{0,x_j} = 1-|x_j|, \end{aligned} \tag{S.62}$$

where we have set the measurement outcomes as $a_1 = 1$, $a_2 = 0$ and $a_3 = -1$ respectively. One can see that $\hat{p}_i(\vec{x})$ can be viewed as the average of N independent identical measurements. By defining $y_i \equiv \delta_{a_i,x}$ with x the outcome of single measurement, we have

$$\Sigma(\hat{p}_1,\hat{p}_2) = \frac{1}{N}\Sigma(y_1,y_2). \tag{S.63}$$

The right-hand side of the above equation, i.e. $\Sigma(y_1,y_2)$, can be extracted from the experimental result $\{y_i^{(1)},\cdots,y_i^{(N)}\}$ via the well-known Bessel's formula as

$$\Sigma(y_1,y_2)_{ij} \equiv \mathrm{Cov}(y_i,y_j) = \frac{1}{N-1}\sum_{k=1}^{N}(y_i^{(k)}-\bar{y}_i)(y_j^{(k)}-\bar{y}_j), \tag{S.64}$$

where

$$\bar{y}_i = \frac{1}{N}\sum_{k=1}^{N} y_i^{(k)}. \tag{S.65}$$

Therefore, we can experimentally extract $\Sigma(\hat{k}_1,\hat{k}_2)$ from $\partial(\hat{\theta},\hat{\varphi})/\partial(\hat{p}_1,\hat{p}_2)$ and the covariance matrix $\Sigma(\hat{p}_1,\hat{p}_2)$.

E. Feasibility of extending experimental approaches to many-body systems

Here we discuss the feasibility of extending our current experimental approaches to many-body quantum systems. The emulated two-dimensional Chern insulator in our paper as well as other topological models can be simulated in ultra-cold atom

systems [13, 14]. Consequently, the ultra-cold atom system in optical lattices, being a highly controllable and flexible many-body system, offers a promising avenue for this extension. Central to our methodology are the optimized positive operator-valued measurements (POVM) for quantum multi-parameter estimation. These techniques have enabled us to effectively probe and characterize the metrological potential in single-particle topological regimes. We would like to point out that the methods used in our experiment, such as the implementation of POVMs in momentum space, have the potential to be extended to the ultra-cold atom system.

Specifically, in ultra-cold atom systems, with the aid of two different internal (pseudospin) states of the atoms, denoted as $|a\rangle$ and $|b\rangle$, we can construct the Bloch Hamiltonian in the $\{|a\rangle, |b\rangle\}$ space [15]. Routine techniques, such as spin-dependent time-of-flight (TOF), allow us to measure the spin-dependent crystal momentum distributions, $n_{a,b}(\boldsymbol{k})$, and thus the population $P_{a,b} = n_{a,b}(\boldsymbol{k})/[n_a(\boldsymbol{k}) + n_b(\boldsymbol{k})]$ in the two internal states [14–17]. This enables the implementation of spin projections along the $\hat{z}$-direction in momentum space. A rapid Raman pulse during TOF allows us to rotate the atomic states, providing NMR-like control over atomic states, which in turn enables spin projection along any direction [15]. Additionally, the implementation of three-output POVM in an ultra-cold atom system can be achieved by utilizing an extra internal state, $|c\rangle$, and coherently controlling these internal states, which can be achieved in the experiment [13].

It is important to note that although the emulated two-dimensional Chern insulator described in our paper represents a many-body system, it is based on the independent electron approximation, which does not directly consider the interactions between electrons. However, general many-body systems inherently possess greater complexity due to interactions among a large number of particles. These interactions can lead to phenomena such as entanglement, nonlocality, and criticality, which pose significant challenges in terms of scalability and control. Therefore, we acknowledge the numerous challenges involved in extending our methods to general many-body systems. These challenges include implementing POVMs for many-body states, ensuring the precision of measurement, and interpreting and processing data within the complex context of many-body states.

F. Metrological characterization of topological bands

Topological invariant, quantum volume and metrological potential

In this section, we briefly review the relation between the topological invariants, the quantum volume and the bound for quantum metrology, which is related to our experiments. We refer further details to Ref.[3]. It is important to note that the left-hand side of the metric-curvature correspondence relation in Eq. (S.6) integrates to an integer topological invariant of the occupied Bloch band,

$$\mathrm{Ch}_n = \frac{1}{n!}\left(\frac{i}{2\pi}\right)^n \int_{\mathbb{T}^{2n}} \mathrm{Tr}\left(\Omega^n\right) \in \mathbb{Z}, \tag{S.66}$$

which is known as the n-th Chern number. This topological invariant is central in the classification of Chern insulators. The metric-curvature correspondence relation in Eq. (S.6) implies the inequality (see Eq.(18) in Ref. [3])

$$|\mathrm{Ch}_n| \leqslant \frac{(2n)!}{2^{n(n-1)+1} n! \pi^n} \mathrm{vol}_g\left(\mathbb{T}^{2n}\right), \tag{S.67}$$

where the *quantum volume* (also known as the complexity of the band) is defined as [3]

$$\mathrm{vol}_g\left(\mathbb{T}^{2n}\right) = \int_{\mathbb{T}^{2n}} \sqrt{\det(g)} \mathrm{d}^{2n} k. \tag{S.68}$$

We note that the equality in the above equation is satisfied provided $\mathrm{sgn}(d\vec{n})$ is constant (everywhere where it is meaningful, i.e., where $\sqrt{\det(g)} \neq 0$), or, equivalently, if the function on the left-hand side of Eq. (S.6) does not change sign. We can see that quantum volume is an important quantity related to quantum topology from Eq. (S.67).

From the quantum SLD-CRB in Eq. (S.4), we can establish a relationship between the performance of quantum parameter estimation and the quantum volume as [3]

$$\int_{\mathbb{T}^d} \left(\sqrt{\det \Sigma(\hat{\boldsymbol{k}})}\right)^{-1} \mathrm{d}\boldsymbol{k} \leqslant 2^d N^{d/2} \mathrm{vol}_g\left(\mathbb{T}^d\right), \tag{S.69}$$

where the left-hand side is a quantity that characterizes the performance of global sensing [18]. Furthermore, in the case of Eq. (S.67) taking the equal sign, we can establish a fundamental connection between quantum parameter estimation and

topological invariants

$$\int_{\mathbb{T}^d}\left(\sqrt{\det\Sigma(\hat{\boldsymbol{k}})}\right)^{-1}\mathrm{d}\boldsymbol{k}\leqslant N^{d/2}\frac{2^{n(n+1)+1}n!\pi^n}{(2n)!}\left|\mathrm{Ch}_n\right|. \tag{S.70}$$

In the two-dimensional case, the above equation becomes

$$M_p\equiv\frac{1}{N}\int_{\mathbb{T}^2}\left(\sqrt{\det\Sigma(\hat{\boldsymbol{k}})}\right)^{-1}\mathrm{d}k_1\mathrm{d}k_2\leqslant 4\,\mathrm{vol}_g\left(\mathbb{T}^2\right)=2\int_{\mathbb{T}^2}\left|\Omega_{12}(\boldsymbol{k})\right|\mathrm{d}k_1\mathrm{d}k_2. \tag{S.71}$$

And if Eq. (S.67) takes the equal sign, we can get

$$M_p=\int_{\mathbb{T}^2}\left(\sqrt{\det\Sigma(\hat{\boldsymbol{k}})}\right)^{-1}\mathrm{d}k_1\mathrm{d}k_2\leqslant 4\pi N\left|\mathrm{Ch}_1\right|. \tag{S.72}$$

The above discussions focus on the systems that has no chiral symmetry, while similar conclusions hold for the systems with chiral symmetry. We now set $d=D-1=2n-1$ for some integer $n>0$. In this case, $H(\boldsymbol{k})$ is also generically gapped but chiral symmetry is now present. In this case, the topological invariant of the occupied Bloch band is the winding number ν, which completely classifies the topological phase

$$\nu=(-1)^{n-1}\left(\frac{i}{2\pi}\right)^n\frac{(n-1)!}{(2n-1)!}\int_{\mathbb{T}^{2n}}\mathrm{Tr}\left[\left(q^{-1}dq\right)^{2n-1}\right]\in\mathbb{Z}. \tag{S.73}$$

Similar to Eq. (S.67), we have an inequality for the winding number (see Eq.(37) in Ref. [3])

$$|\nu|\leqslant\frac{(n-1)!}{2^{\frac{1}{2}(n-1)(2n-5)}\pi^n}\mathrm{vol}_g\left(\mathbb{T}^{2n-1}\right). \tag{S.74}$$

Again, we note that the equality is satisfied when $\mathrm{sgn}(d\vec{n})$ is constant (for $\sqrt{\det(g)}\neq 0$)). In this case, we can get an equation similar to Eq. (S.70) when Eq. (S.74) takes the equal sign

$$\int_{\mathbb{T}^{2n-1}}\left(\sqrt{\det\Sigma(\hat{\boldsymbol{k}})}\right)^{-1}\mathrm{d}^{2n-1}k\leqslant N^{d/2}\frac{2^{\frac{1}{2}(n-1)(2n-1)+1}\pi^n}{(n-1)!}|\nu|. \tag{S.75}$$

Divergence of Uncertainty Volume in Fig. 4(d)

According to the Berry curvature bound in Eq. (S.8) [i.e., Eq. (5) in the main text], it is evident that at points where the Berry curvature vanishes, the uncertainty volume will diverge. The significant variations of the uncertainty volume near $k_2=\pi/4$ in Fig. 4(d) of the main text is just due to the zero Berry curvature. In topologically trivial regimes, where the Chern number is zero, the integral of the Berry curvature across the entire Brillouin zone cancels out. For systems where the Hamiltonian varies smoothly with respect to the parameters, the Berry curvature is generally a continuous function across the parameter space. This continuity necessitates the existence of boundaries where the Berry curvature is zero in topologically trivial regime, leading to the divergence of the uncertainty volume and the pronounced changes observed with k. It is also important to note that for two-dimensional two-band models, there must exist a point in the Brillouin zone where the Berry curvature is zero, even if the Chern number is nonzero (see Theorem 3 in Ref. [19]). Additionally, the noticeable change in the uncertainty volume at $k_2=0,\pi$ in Fig. 4(d) is attributed to the specific symmetric POVM (sPOVM) utilized in our experiments, which does not perform well around these parameter points within the topologically trivial area.

To elucidate further, we consider the specific model presented in the Eq. (1) of the main text. Given that our chosen trajectory in Fig. 4(d) has $k_1=0$, the Hamiltonian along this path simplifies to

$$H(\boldsymbol{k})=\boldsymbol{d_k}\cdot\boldsymbol{\sigma}=\begin{bmatrix}\sin k_1\\ \sin k_2\\ M-\cos k_1-\cos k_2\end{bmatrix}\cdot\boldsymbol{\sigma}=\begin{bmatrix}0\\ \sin k_2\\ M-1-\cos k_2\end{bmatrix}\cdot\boldsymbol{\sigma} \tag{S.76}$$

with $k_1=0$, varying k_2 traces the terminal points of $\boldsymbol{d_k}$ as a circle in the d_y-d_z plane, centered at $(0,M-1)$ with a radius of 1, as depicted in the Fig. S.5. The topologically trivial regime corresponds to the case where the circle does not enclose the origin, as shown in Fig. S.5(b). The excited state $|\psi(\boldsymbol{k})\rangle$ of the $H(\boldsymbol{k})$ corresponds to the unit vector $\boldsymbol{n_k}=\boldsymbol{d_k}/|\boldsymbol{d_k}|$ on the Bloch

sphere. For a two-level Hamiltonian in the form of $H(\boldsymbol{k}) = \boldsymbol{d_k} \cdot \boldsymbol{\sigma}$, the Berry flux is related to the solid angle subtended by the unit vector $\boldsymbol{n_k}$, so that the Berry curvature of $|\psi(\boldsymbol{k})\rangle$ takes the form [20]

$$\Omega_{12}(\boldsymbol{k}) = -\frac{1}{2}(\partial_{k_1}\boldsymbol{n_k} \times \partial_{k_2}\boldsymbol{n_k}) \cdot \boldsymbol{n_k} \tag{S.77}$$

In the topologically trivial case, when $\boldsymbol{d_k}$ is tangent to the circle, as represented by $\boldsymbol{d'_k}$ in Fig. S.5(b), the derivative $\partial_{k_2}\boldsymbol{n_k}$ equals zero. This indicates that at this point, the state $|\psi(\boldsymbol{k})\rangle$ does not vary with k_2, leading to a zero Berry curvature and hence a divergent uncertainty volume. We have plotted the Berry curvature $\Omega_{12}(\boldsymbol{k})$ as a function of k_2 for the topologically trivial point $M = 2.5$, illustrated in Fig. S.5(c), where the Berry curvature is zero at the point k'_2.

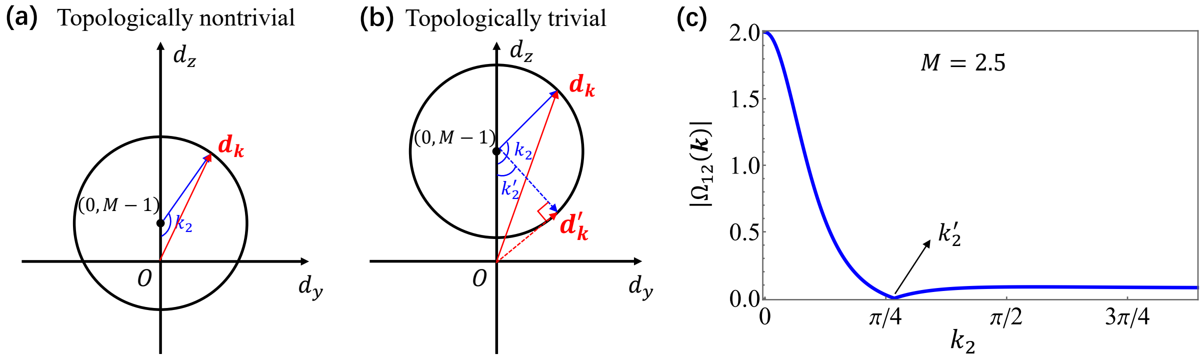

FIG. S.5. Topology of the two-band Chern insulator and the Berry curvature in parameter space. (a-b) terminal points of $\boldsymbol{d_k}$ with $k_1 = 0$ for topologically different regimes. (c) The Berry curvature along the trajectory of Fig. 4(d) in the main text.

G. Independent experimental measurement of quantum geometric tensor

The quantum metric data using in the main text are extracted by measuring the fidelity between neighbouring quantum state in parameter space [21]. The fidelity between neighboring quantum states in parameter space is given by

$$|\langle\psi(\boldsymbol{k}+d\boldsymbol{k})|\psi(\boldsymbol{k})\rangle|^2 = 1 - \sum_{i,j} g_{ij}(\boldsymbol{k}) dk_i dk_j + O(dk_i dk_j dk_l), \tag{S.78}$$

where $g_{ij}(\boldsymbol{k})$ is the quantum metric in momentum space. So we can prepare the state $|\psi(\boldsymbol{k}+d\boldsymbol{k})\rangle$ and measure the overlap between $|\psi(\boldsymbol{k}+d\boldsymbol{k})\rangle$ and $|\psi(\boldsymbol{k})\rangle$. By changing $d\boldsymbol{k}$ in a range that $O(dk_i dk_j dk_l)$ is negligible and repeat the above measurement, we can extract the quantum metric by fitting to the function in Eq. (S.78).

As for the Berry curvature, there have existed several efficient methods that can help to directly extract it, such as the methods of weak parametric modulation [22–24] and the quasiadiabatic dynamical response [25, 26]. In our experiment, we independently measure the Berry curvature using the weak-parametric-modulation method [23], which provides the Berry curvature data for Fig.2 in the main text. Generally, this method can be exploited to extract the quantum metric as well, and thus the complete quantum geometric tensor as well [23].

* These authors contributed equally.
† yaomingchu@hust.edu.cn
‡ bruno.mera.c5@tohoku.ac.jp
§ fnu20@cam.ac.uk
¶ nathan.goldman@ulb.be
** jianmingcai@hust.edu.cn

[1] M. Yu, Y. Liu, P. Yang, M. Gong, Q. Cao, S. Zhang, H. Liu, M. Heyl, T. Ozawa, N. Goldman, and J. Cai, Quantum Fisher information measurement and verification of the quantum Cramér–Rao bound in a solid-state qubit, npj Quantum Inf. **8**, 56 (2022).
[2] J. Liu, H. Yuan, X.-M. Lu, and X. Wang, Quantum Fisher information matrix and multiparameter estimation, J. Phys. A Math. Theor. **53**, 023001 (2020).

[3] B. Mera, A. Zhang, and N. Goldman, Relating the topology of Dirac Hamiltonians to quantum geometry: When the quantum metric dictates Chern numbers and winding numbers, SciPost Phys. **12**, 18 (2022).
[4] J. K. Asbóth, L. Oroszlány, and A. Pályi, A short course on topological insulators, Lecture notes in physics **919**, 166 (2016).
[5] T. Ozawa and B. Mera, Relations between topology and the quantum metric for Chern insulators, Phys. Rev. B **104**, 045103 (2021).
[6] R. Demkowicz-Dobrzański, W. Górecki, and M. Guţă, Multi-parameter estimation beyond quantum Fisher information, J. Phys. A Math. Theor. **53**, 363001 (2020).
[7] F. Albarelli, M. Barbieri, M. Genoni, and I. Gianani, A perspective on multiparameter quantum metrology: From theoretical tools to applications in quantum imaging, Phys. Lett. A **384**, 126311 (2020).
[8] A. Carollo, B. Spagnolo, A. A. Dubkov, and D. Valenti, On quantumness in multi-parameter quantum estimation, Journal of Statistical Mechanics: Theory and Experiment **2019**, 094010 (2019).
[9] C. Li, M. Chen, and P. Cappellaro, A geometric perspective: experimental evaluation of the quantum Cramer-Rao bound, arXiv:2204.13777 (2022).
[10] J. M. Renes, R. Blume-Kohout, A. J. Scott, and C. M. Caves, Symmetric informationally complete quantum measurements, J. Math. Phys. **45**, 2171 (2004).
[11] N. Li, C. Ferrie, J. A. Gross, A. Kalev, and C. M. Caves, Fisher-symmetric informationally complete measurements for pure states, Phys. Rev. Lett. **116**, 180402 (2016).
[12] C. A. Fuchs, M. C. Hoang, and B. C. Stacey, The SIC question: History and state of play, Axioms **6**, 21 (2017).
[13] N. Goldman, J. C. Budich, and P. Zoller, Topological quantum matter with ultracold gases in optical lattices, Nature Physics **12**, 639 (2016).
[14] N. R. Cooper, J. Dalibard, and I. B. Spielman, Topological bands for ultracold atoms, Rev. Mod. Phys. **91**, 015005 (2019).
[15] E. Alba, X. Fernandez-Gonzalvo, J. Mur-Petit, J. K. Pachos, and J. J. Garcia-Ripoll, Seeing topological order in time-of-flight measurements, Phys. Rev. Lett. **107**, 235301 (2011).
[16] P. Hauke, M. Lewenstein, and A. Eckardt, Tomography of band insulators from quench dynamics, Phys. Rev. Lett. **113**, 045303 (2014).
[17] Z. Wu, L. Zhang, W. Sun, X.-T. Xu, B.-Z. Wang, S.-C. Ji, Y. Deng, S. Chen, X.-J. Liu, and J.-W. Pan, Realization of two-dimensional spin-orbit coupling for Bose-Einstein condensates, Science **354**, 83 (2016).
[18] V. Montenegro, U. Mishra, and A. Bayat, Global sensing and its impact for quantum many-body probes with criticality, Phys. Rev. Lett. **126**, 200501 (2021).
[19] B. Mera and T. Ozawa, Kähler geometry and Chern insulators: Relations between topology and the quantum metric, Phys. Rev. B **104**, 045104 (2021).
[20] M. Z. Hasan and C. L. Kane, Colloquium: Topological insulators, Rev. Mod. Phys. **82**, 3045 (2010).
[21] M. Yu, D. Li, J. Wang, Y. Chu, P. Yang, M. Gong, N. Goldman, and J. Cai, Experimental estimation of the quantum Fisher information from randomized measurements, Phys. Rev. Research **3**, 043122 (2021).
[22] T. Ozawa and N. Goldman, Extracting the quantum metric tensor through periodic driving, Phys. Rev. B **97**, 201117 (2018).
[23] M. Yu, P. Yang, M. Gong, Q. Cao, Q. Lu, H. Liu, S. Zhang, M. B. Plenio, F. Jelezko, T. Ozawa, N. Goldman, and J. Cai, Experimental measurement of the quantum geometric tensor using coupled qubits in diamond, Natl. Sci. Rev. **7**, 254 (2020).
[24] M. Chen, null null, C. Li, G. Palumbo, Y.-Q. Zhu, N. Goldman, and P. Cappellaro, A synthetic monopole source of Kalb-Ramond field in diamond, Science **375**, 1017 (2022).
[25] V. Gritsev and A. Polkovnikov, Dynamical quantum Hall effect in the parameter space, Proceedings of the National Academy of Sciences **109**, 6457 (2012).
[26] P. Roushan, C. Neill, Y. Chen, M. Kolodrubetz, C. Quintana, N. Leung, M. Fang, R. Barends, B. Campbell, Z. Chen, *et al.*, Observation of topological transitions in interacting quantum circuits, Nature **515**, 241 (2014).
